# Is myocardial fibrosis appropriately assessed by calibrated and 2D strain derived integrated backscatter?

**DOI:** 10.1186/s12947-023-00311-x

**Published:** 2023-08-12

**Authors:** Maria Rita Lima, João Abecasis, Rita Reis Santos, Sérgio Maltês, Pedro Lopes, António Ferreira, Regina Ribeiras, Maria João Andrade, Miguel Abecasis, Victor Gil, Sância Ramos, Nuno Cardim

**Affiliations:** 1 Cardiology Department, Hospital de Santa Cruz, Centro Hospitalar Lisboa Ocidental, Lisbon, Portugal; 2 Nova Medical School, Lisbon, Portugal; 3 Cardiac Surgery Department, Hospital de Santa Cruz, Centro Hospitalar de Lisboa Ocidental, Lisbon, Portugal; 4 Hospital da Luz, Lisbon, Portugal; 5 Pathology Anatomy Department, Hospital de Santa Cruz, Centro Hospitalar de Lisboa Ocidental, Lisbon, Portugal

**Keywords:** Ultrasound calibrated integrated backscatter, Myocardial fibrosis, Collagen volume fraction, Cardiac magnetic resonance, Aortic stenosis

## Abstract

**Aims:**

Increased collagen content of the myocardium modifies tissue reflectivity and integrated backscatter (IBS) indexes are suggested as markers of myocardial fibrosis (MF). We sought to assess the correlation between calibrated (c) IBS and bidimensional (2D) strain derived IBS with left ventricular (LV) MF in patients with severe aortic stenosis (AS).

**Methods and results:**

We made a prospective observational cohort study including 157 patients with severe AS referred for surgical aortic valve replacement (AVR), with complete preoperative transthoracic echocardiography, cardiac magnetic resonance (CMR) and endomyocardial biopsy (EMB) obtained from the anterior basal septum at the time of surgery. Two groups of 30 patients were specifically evaluated, with and without late gadolinium enhancement (LGE) at CMR. IBS was obtained at QRS peak from both parasternal long axis (PLAX) and apical-three-chamber (AP3C) views and measured in decibels (dB). Whole-cardiac cycle IBS at basal anterior septum was obtained from 2D longitudinal strain. Correlation analysis of reflectivity indexes was performed with global and segmental (anterior basal septum) values of native T1 and extracellular volume (ECV), and EMB collagen volume fraction (CVF) (Masson´s Trichrome). IBS values were compared in both group of patients (LGE + vs. LGE –). 60 patients (74 [36–74] years, 45% male) with high gradient (mean gradient: 63 ± 20mmHg), normal flow (45 ± 10mL/m^2^) AS and preserved left ventricular ejection fraction (60 ± 9%) were included. Basal septum cIBS was − 17.45 (–31.2–10.95) and − 9.17 ± 9.45dB from PLAX and A3C views, respectively. No significant correlations were found between IBS and both non-invasive CMR tissue characterization and CVF: median MF of 9.7(2.1–79.9)%. Acoustic indexes were not significantly different according to the presence of pre-operative LGE.

**Conclusion:**

In this group of patients with classical severe AS, IBS reflectivity indexes are of no added value to discriminate the presence of MF.

**Graphical Abstract:**

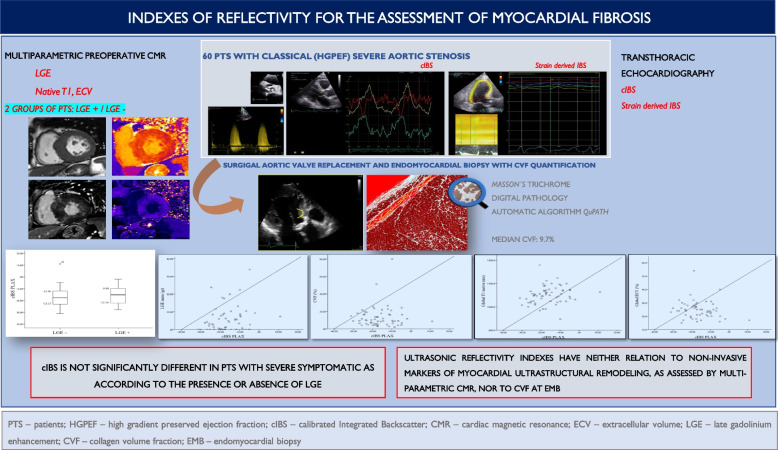

**Supplementary Information:**

The online version contains supplementary material available at 10.1186/s12947-023-00311-x.

## Introduction

Degenerative aortic stenosis (AS) is the most common valvular heart disease, characterized by the progressive narrowing of the aortic valve and concomitant left ventricular (LV) adaptation to chronic pressure overload [1]. Ultrastructural myocardial remodeling is an early process occurring for less than severe stages of the disease [2, 3]. Such remodeling may include a variable degree of myocardial fibrosis (MF), a recognized and independent marker of LV decompensation, heart failure development and clinical outcomes in this setting, even for asymptomatic patients with severe AS [4–6].

Endomyocardial biopsy (EMB) remains the gold standard method to assess cardiomyocyte adaptation and myocardium extracellular remodeling. Collagen volume fraction (CVF), as provided from histomorphometry analysis, is correlated with myocardial collagen content and is a recognized tool for the quantitative assessment of MF [7]. However, this analysis relies on an invasive and costly procedure, not capturing the global myocardial involvement and with inconsistent clinical correlation in this context [8].

Currently, cardiac magnetic resonance (CMR) is progressing as the imaging modality of choice for the non-invasive detection and quantification of LV fibrosis in a wide range of clinical scenarios [9]. It may provide valuable information regarding myocardial tissue composition, cellular and extracellular compartments´ expansion, and specifically diffuse and replacement fibrosis, on top of accurate measurements of LV volumes, mass, and ejection fraction (EF), deemed gold standard. Despite variable among studies, the correlation between the quantification of fibrosis by CMR and invasive CVF has been demonstrated [10]. Moreover, a recent meta-analysis found that late gadolinium enhancement (LGE), native myocardial T1 and extracellular volume (ECV) derived values, as yielded by comprehensive CMR protocols, are independent predictors of the outcome and occurrence of cardiovascular events in patients with severe AS [6]. Even so, CMR is not part of the daily routine workup study of patients with severe AS, despite its valuable information upon myocardial adaptation in this setting.

Ancillary transthoracic echocardiography (TTE) studies tried to assess myocardial reflectivity properties by ultrasonic integrated backscatter (IBS) signal [11–14]. As extracellular matrix collagen seems to be the primary determinant of scattering in the myocardium, echocardiographic IBS seemed an attractive tool for tissue characterization, as a measurement of CVF and fibrosis. In this way, a single non-invasive echocardiographic study would provide myocardial ultrastructural detail, beyond both functional LV and valve assessment. Yet, previous small-sample reports used distinct ultrasound modalities (e.g. M-Mode, 2D imaging, speckle tracking) and protocols to assess IBS, resulting in poor correlations between TTE findings and MF at EMB [13, 15]. Additionally, impedance quantification was mostly laborious and seemed dependent on acquisition settings (imaging depth, gain settings, region of interest area, temporal resolution) for specific modalities, vendors, and machines. At the end, poor correlations have been found with histology for patients with less than extensive grades of MF [16–18], with paucity of data supporting its clinical application.

Hence, our aim was to assess the correlation between echocardiographic derived IBS and LV myocardial fibrosis in a group of patients with severe symptomatic AS, as evaluated by both non-invasive CMR and myocardial biopsy.

## Methods

### Study population

From a cohort of 157 consecutive patients with severe symptomatic AS referred and accepted for aortic valve replacement (AVR) surgery at our tertiary centre between April 2019 and January 2022, we randomly selected 30 patients with and 30 patients without LV LGE at pre-operative CMR for the analysis in two groups, as specified in Supplementary Fig. 1. This was part of a correlation research protocol involving both pre- and post-operative LV structural and functional assessment by multimodality imaging and myocardial histopathology study from EMB, specially addressing cardiomyocyte adaptation and extracellular matrix remodeling and fibrosis. AS was defined according to European guidelines on valvular heart disease [19]. Study approval was granted by the ethical committee of Nova Medical School University (number 61/2018/CEFCM) conforming to the principles of the Helsinki declaration. All participants gave written informed consent before inclusion. Exclusion criteria are detailed at Supplementary material – Methods. Two groups of patients with complete TTE, CMR and EMB were constituted. IBS derived data were compared in both groups of patients and correlated with both non-invasive CMR measurements and myocardial fibrosis at histopathology.

### Clinical data and study design

Clinical parameters (demographic, major cardiovascular risk factors and symptomatic status), 12-lead ECG and TTE were collected at study inclusion before AVR. CMR was performed within 2 weeks after patient inclusion alongside blood sample for haematocrit (Htc), creatinine, high-sensitivity cardiac troponin I (hsTnI) and N-terminal pro b-type natriuretic peptide (NT-proBNP). Both TTE and CMR studies were performed within 6 months before AVR. If clinically justified for coronary artery disease exclusion, patients performed a coronary angiography before intervention and coronary revascularization was added to AVR when indicated. Surgical myectomy was concomitantly performed if planned before intervention because of asymmetric septal hypertrophy or at surgeon´s discretion.

### Standard echocardiographic study – evaluation for aortic valve stenosis

All patients underwent a comprehensive TTE by experienced cardiologists before AVR, using commercially available ultrasound systems (Vivid E9; GE Healthcare, Chicago, IL, USA) with a 4D probe (3.5-MHz 2D phased array transducer), in accordance with current guidelines [20–22] (Supplementary Material – Methods). Imaging analysis and measurements were performed on image data stored in the regional image vault and re-examined using EchoPAC version 202 for PC (GE Healthcare, Milwaukee, WI, USA).

#### Calibrated Integrated Backscatter (cIBS)

**Fig. 1:**
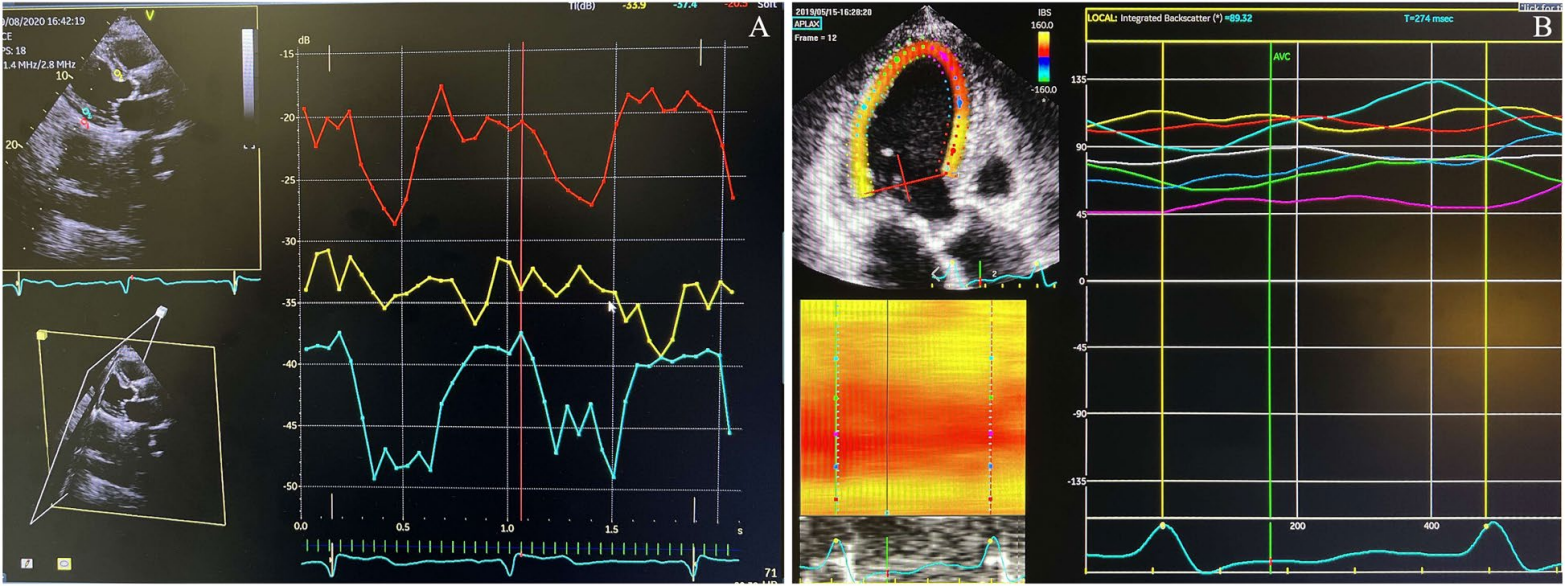
**A** IBS curves from basal anterior septum (yellow curve), inferolateral wall (blue curve) and pericardium (red curve) as determined from PLAX; **B** 2D longitudinal strain derived whole cycle IBS. Red curve – basal anterior septum; darker blue curve – middle segment of the septum; purple curve – apical segment of the septum; green curve – apical segment of the inferolateral wall; lighter blue curve – middle segment of the inferolateral wall; yellow curve – basal inferolateral wall; white curve – mean whole-cycle IBS. AVC – Aortic valve closure; IBS – Integrated backscatter; PLAX – parasternal long-axis view

As previously described [13] and recommended [23], we used bidimensional parasternal long-axis view (PLAX) (averaged profiles over three cardiac cycles), with a frame rate between 50 and 80 frames/s, to estimate IBS. This was performed at EchoPAC for the acquisition of the curves, placing regions of interest (ROI) with equal shape and dimension in the mid-myocardium, avoiding the endocardium, of both basal anterior septum and basal inferolateral wall and in the pericardium, as the reference. ROI position was adjusted as appropriate in each frame to keep the sample in the same area of the myocardium throughout the whole cardiac cycle (Fig. 1A). cIBS was obtained by subtracting the pericardial IBS, expressed in decibels (dB) at end-diastole (QRS peak), from the average of the basal anteroseptal and inferolateral wall IBS. Even considering that accepted measurements are restricted to parasternal views (where the ultrasound beam is perpendicular to the interrogated wall), we tried cIBS assessment, with the same methodology, from apical three chamber (AP3C) view acquisitions at the same LV segments and corresponding basal regions.

Beyond this, we used *Qanalysis* and longitudinal strain imaging at AP3C view to obtain LV myocardial deformation curves and gather average whole-cardiac cycle absolute values of IBS at basal anterior septum (designated as strain-derived IBS) (Fig. 1B). *Qanalysis* is a specific EchoPAC tool in which each operator selects specific LV points, blood-endocardial interface, and the thickness of the ROI for the specific speckle tracking analysis at each cine-loop dataset. Strain-derived IBS is supposed to be correlated with myocardial strain, being a possible non-invasive marker of contractility, more than reflecting ultrastructural composition [15, 24].

### Cardiac magnetic resonance

CMR study was performed at 1.5T equipment (Magnetom Avanto; Siemens Medical Solutions, Erlangen, Germany) using a clinical scan protocol, as previously published [25]. Methods for image acquisition, pre- and post-contrast tissue characterization, post-processing and quantification, are detailed in Supplementary Material – Methods.

For the assessment of reflectivity indexes in both groups of patients, with and without LGE at CMR, we did not specifically perform the analysis according to the presence of LGE at the basal anterior septum. This would be supposed to be closer or coincident to the place of echocardiographic assessment. However, we suspected that this could limit the number of patients in the group with LGE.

### Myocardial fibrosis quantification at Histopathology

EMB samples were obtained either from intraoperative septal biopsy as per protocol design (harvested with a scalpel from the basal interventricular septum, preferably with endocardium included) or from complementary septal myectomy, performed by the surgical team at the time of surgical AVR. In a pre-analytical phase, biopsies were fixed in 10% neutral buffered formalin (*JTBaker*®) for 24 to 48 h at room temperature (20ºC). Formalin fixed, paraffin (*VWR International*, USA) embedded tissues were processed in *Sakura´s “Tissue-Tek VIP”* and cut into 3 μm thick sections. Tissue section adhesion time and temperature were constant for one hour at 70ºC. Sections were stained with Masson´s trichrome for collagen/fibrosis assessment according to a standard protocol. Using a digital microscopic scanner (ScanScope® AT2 brightfield scanner TM, Aperio Technologies, Vista, CA, USA) we obtained whole slide images (WSI), at a resolution of 0.5 μm. These were analysed at a dedicated algorithm specifically developed for brightfield images at the software platform QuPath 0.3.0. For quantification, dense endocardial fibrosis was excluded (Supplementary Fig. 2) and total tissue component was split into fibrosis and a remaining part, containing cardiomyocytes, empty spaces, vessels, and blood cells. All components were assessed in pixels and a final table containing both absolute areas and correspondent proportions were obtained for each tissue sample (Supplementary Fig. 3). Collagen volume fraction was defined as the percentage of fibrosis = (area of fibrosis / area of cardiomyocytes + area of fibrosis) x 100. These measurements were made with no knowledge of the clinical data.

Technical details addressing the quantification algorithm and *QuPath* platform are specified in Supplementary Material – Methods.

### Statistical analysis

Categorical variables were reported as numbers and percentages, and continuous variables as mean ± standard deviations (normal distribution), or as median and interquartile range for variables with skewed distributions. Normal distribution was checked using Shapiro-Wilk test or skewness and kurtosis, as appropriate. Clinical characteristics of the subgroups of interest were compared using the χ2-test and Fisher’s exact test (when applicable) for dichotomous variables; and the student’s t-test or Mann-Whitney U test (when applicable) for continuous variables. Categorical variables were compared by two-tailed chi-square. Pearson or Spearman’s correlation coefficients were used to assess the correlation between global and segmental (anterior basal septum) values of native T1 and ECV, EMB CVF and cIBS within overall cohort and between each group. Inter and intra-observer variability for the quantification of reflectivity indexes was evaluated using Bland-Altman plots [26]. This was performed at the first evaluation by two blinded independent operators and one week later by one of them to assess intra-observer variability.

A two-sided *p*-value < 0.05 was considered statistically significant. The statistical analysis was performed with IBM SPSS Statistics 26.0 (IBM Corp, Armonk, NY, USA).

## Results

### Overall characterization

A total of 60 patients with severe symptomatic AS and appropriate preoperative echocardiographic study, complete comprehensive CMR protocol and septal EMB performed at surgical AVR were included in this analysis. The median age of the patients was 74 [36-74] years, with female predominance (55%). These were all ambulatory patients, mostly with effort dyspnoea or functional impairment on daily activities. Overall clinical and laboratory data are summarized in Table 1. These patients had predominant high gradient, normal flow, preserved EF AS (only 6 patients with LVEF between 40 and 50%). LV concentric remodeling and hypertrophy were the predominant patterns of adaptation as determined by CMR (from LV mass, volumes, and geometric remodeling). Both global and basal anteroseptal native T1 values were significantly above institutional normal T1 values (1049.5 vs. 1071.0, respectively; *p* < 0.001). Patients showed a median LGE mass of 3.0 g (0.0–23.8), exclusively with a mid-myocardial and/or junctional pattern. Median ECV values were within normal range. At EMB, all, except four of the cases, had appropriate endocardial inclusion. All types of myocardial fibrosis (interstitial and perivascular type; microscars) were included for CVF quantification at the automatic algorithm. Pre-operative imaging and histopathology data are presented in Table 2.

**Table 1:** Baseline clinical and laboratory data

Total of patients included: *n* = 60	
**Clinical characteristics**	
Age, years	73.5 (36.0–84.0) (IQR = 8.8)
Male	27 (45%)
BSA, m^2^	1.78 ± 0.17
Hypertension	48 (80.0%)
Diabetes mellitus	17 (28.3%)
Dyslipidemia	34 (56.7%)
Smoking history	13 (21.7%)
Previous stroke	1 (1.7%)
Atrial fibrillation	7 (11.7%)
Previous pacemaker implantation	0 (0.0%)
NYHA functional class	
I	1 (0.8%)
II	42 (35.3%)
III	17 (14.3%)
Anginal symptoms	13 (10.9%)
Syncope	14 (11.8%)
**Laboratory results**	
Htc, %	39.3 ± 3.1
Creatinin, mg/dL	0.91 (0.58–2.26)
Estimated glomerular filtration rate, mL/min	71.47 ± 26.09
NT-proBNP, pg/mL	561.0 (41.0–22664.0)
Troponin I, ng/L	16.2 (0-115)

Values are median (interquartile range); n (%); mean ± standard deviation

*BSA* Body surface area, *Htc* Haematocrit, *NT–proBNP* N–terminal B–type natriuretic peptide, *NYHA* New York Heart Association

**Table 2:** Pre-operative imaging and histopathology data

Total of patients included: *n* = 60	
**Echocardiography**	
Aortic valve area, cm^2^	0.67 ± 0.19
Maximum aortic gradient, mmHg	99.89 ± 30.58
Mean aortic gradient, mmHg	63.1 ± 20.2
Stroke volume index, mL/m^2^	43.97 ± 9.31
LV indexed mass, g/m^2^	141.73 (67.27–360.83)
Maximum septal thickness, mm	16.0 ± 2.8
LVEF, %	57.01 ± 8.32
Global longitudinal strain, %	–14.57 ± 3.77
PLAX cIBS, dB	–17.45 (–31.2–10.95)
AP3C cIBS, dB	–9.17 ± 9.45
AP3C, strain derived IBS, dB	113.99 ± 22.59
**Cardiac Magnetic Resonance**	
LV indexed mass, g/m^2^	76.2 ± 23.3
LVEDV, mL	148.9 ± 37.6
Geometric remodeling, g/mL	0.93 ± 0.22
LVEF, %	59.6 ± 9.0
Delayed enhancement, g	3.03 (0–23.8)
Delayed enhancement, % of mass	2.50 (0.0–21.0)
Global native T1, ms	1049.5 (947.0–1179.0)
Basal anteroseptal native T1, ms	1071.0 (965.0–10130.0)
Global ECV, %	24.0 (15.0–54.0)
Basal anteroseptal ECV, %	22.0 (13.1–44.2)
**Histopathology at EMB**	
Fibrosis, % (CVF)	9.7 (6.15–16.7)

Values are median (interquartile range); mean ± standard deviation

*AP3C* Apical 3-chamber view, *cIBS* Calibrated integrated backscatter, *CVF* Collagen volume fraction, *dB* Decibel, *ECV* Extracellular volume, *EDV* End-diastolic volume, *EF* Ejection fraction, *EMB* Endomyocardial biopsy, *LV* Left ventricle, *PLAX* Parasternal long axis view

### Integrated backscatter in both group of patients, LGE + vs. LGE 

Clinical and imaging data, including IBS indexes, in patients with and without LGE at preoperative CMR are depicted in Table 3. We did not find significant differences in both groups of patients in what concerns clinical and imaging data, namely regarding AS severity and both LV remodeling and additional tissue characterization. IBS parameters neither significantly differed in patients with and without LGE (Boxplots at Supplementary Material - Results). Fibrosis content at histopathology was similar in both group of patients.

**Table 3:** Clinical and imaging data in both group of patients, with and without LGE at pre-operative CMR

	*LGE -*	*LGE +*	*P-value*
Age, years	74.0 (36.0–80.0)	73.0 (47.0–84.0)	0.689
Male	11 (36.6%)	16 (53.3%)	0.194
Hypertension	23 (76.6%)	25 (83.3%)	0.519
Diabetes	5 (16.6%)	12 (40.0%)	**0.045**
Atrial fibrillation	3 (10.0%)	4 (13.3%)	0.999
Estimated glomerular filtration rate, mL/min	72.09 ± 30.71	70.84 ± 20.99	0.854
Nt-proBNP, pg/mL	695.0 (41.0–22664.0)	583.0 (102.0–7493.0)	0.395
Troponin I, ng/L	11.0 (0.0-115.0)	12.5 (6.0–42.0)	0.859
**Echocardiography**			
Aortic valve area, cm^2^	0.64 ± 0.17	0.67 ± 0.19	0.390
Mean aortic gradient, mmHg	64.71 ± 19.37	65.72 ± 21.29	0.857
Maximum septal thickness, mm	15.90 (10.70–23.00)	15.86 (12.00–22.34)	0.120
LVEF, %	57.43 ± 6.61	56.60 ± 9.84	0.740
GLS, %	–15.16 ± 3.44	–13.94 ± 4.07	0.233
Basal septum longitudinal strain, %	–12.4 ± 6.4	–9.9 ± 6.7	0.141
**Integrated backscatter**			
PLAX cIBS, dB	–17.75 (–31.2–10.95)	–14.45 (–27.8– − 2.05)	0.290
AP3C cIBS, dB	–11.28 ± 7.91	–8.13 ± 9.72	0.189
AP3C, strain derived IBS, dB	110.55 ± 21.22	117.43 ± 23.83	0.241
**Cardiac Magnetic Resonance**			
LV indexed mass, g/m^2^	74.17 ± 24.97	82.49 ± 23.47	0.316
LVEDV, mL	148.53 ± 35.09	149.30 ± 40.60	0.938
Geometric remodeling, g/mL	0.88 ± 0.20	0.97 ± 0.23	0.121
LVEF, %	50.03 ± 7.04	59.50 ± 11.06	0.800
Delayed enhancement, g	NA	6.78 (0.00–33.90)	**< 0.001**
Delayed enhancement, % of mass	NA	6.00 (0.00–21.00)	**< 0.001**
Global native T1, ms	1057.5 (991.0–1179.0)	1047.0 (947.0–1099.0)	0.549
Basal anteroseptal native T1, ms	1066 (1016–1372)	1071 (965–1329)	0.387
Global ECV, %	25.0 (15.0–40.0)	23.0 (16.0–54.0)	0.172
Basal anteroseptal ECV, %	22.45 (13.10–44.20)	21.25 (14.20–32.40)	0.207
**Histopathology at EMB**			
Fibrosis, % (CVF)	9.60 (3.30–51.20)	10.20 (2.10–79.90)	0.796

Values are median (interquartile range); n (%); mean ± standard deviation. Bold *P*-values are statistically significant. *LGE* Late gadolinium enhancement, *NA* Not applicable. Other abbreviations as in Table 2

### Integrated backscatter: correlations with invasive and non-invasive assessment of fibrosis

At Bland-Altman plots, the mean difference of IBS measurements for the same observer was not statistically different. The results were similar for cIBS for both echocardiographic windows and strain derived IBS. Likewise, interobserver variability was negligible except for 2D strain derived mean IBS (Supplementary Material – Results).

cIBS values were significantly lower when calculated from AP3C view [–9.17 ± 9.45 vs. − 17.45 (–31.2–10.95), *p* < 0.001] despite having good correlation between them (Supplementary Material – Results). Strain derived anteroseptal IBS was significantly different from conventional IBS estimation (mean anteroseptal strain derived IBS = 113.99 ± 22.59dB versus mean anteroseptal IBS at PLAX = − 35.85 ± 6.90dB and IBS at AP3C = − 27.16 ± 6.53dB, *p* < 0.001 for both).

We were not able to detect significant correlations between IBS data, as assessed from distinct echocardiographic windows and methodologies, and distinct CMR tissue characterization parameters and CVF from histopathology (Table 4), except for the absolute value of IBS from basal anterior septum, as derived from PLAX, which was weakly correlated to LGE mass (Table 4), and cIBS, also with slight correlation to global myocardial native T1 (Supplementary Material – Results).

**Table 4:** Correlations between IBS measurements and global and regional longitudinal strain, CMR tissue characterization parameters and histopathology CVF

	PLAX ivsIBS (r)	P	PLAX cIBS (r)	P	AP3C cIBS (r)	P	AP3C ivsIBS (r)	P
**Overall population, ** ***n*** **= 60**								
GLS	0.146	0.267	0.229	0.078	0.004	0.976	0.009	0.945
ivsLS	-0.158	0.228	0.114	0.385	0.048	0.714	-0.138	0.292
Global T1	0.105	0.423	0.299	0.020	0.131	0.317	-0.003	0.982
ivsT1	-0.021	0.875	0.165	0.208	0.061	0.641	-0.046	0.727
Global ECV	0.034	0.798	–0.184	0.167	–0.141	0.293	0.013	0.925
ivsECV	–0.140	0.286	–0.076	0.571	–0.024	0.859	0.098	0.457
LGE	0.047	0.720	0.183	0.163	0.198	0.130	-0.146	0.265
CVF	-0.249	0.055	-0.067	0.611	0.122	0.354	-0.189	0.149
**LGE +,** ***n* = 30 **								
GLS	0.494	0.060	0.310	0.096	-0.063	0.739	0.144	0.447
ivsLS	-0.004	0.985	0.303	0.103	-0.065	0.735	-0.001	0.998
Global T1	0.120	0.529	0.093	0.626	-0.144	0.447	0.202	0.283
ivsT1	0.267	0.155	0.096	0.616	0.069	0.716	0.275	0.141
Global ECV	0.160	0.417	–0.315	0.102	0.027	0.890	0.078	0.692
ivsECV	–0.028	0.882	–0.166	0.381	0.188	0.321	0.203	0.282
LGE	0.418	**0.021**	0.233	0.215	0.045	0.812	–0.012	0.951
CVF	–0.180	0.341	–0.054	0.776	0.101	0.597	–0.116	0.541

Bold *P*-values are statistically significant; *GLS* Global longitudinal strain; *ivs* basal interventricular septum; Other abbreviations as in Tables 2 and 3

### Global and regional longitudinal strain: correlation with IBS indexes, CMR tissue characterization and CVF

We found no significant correlations between global and regional (basal anterior septum) longitudinal 2D strain and IBS indexes (Table 4). Except for a weak significant correlation between GLS and LGE mass, there were no significant correlations between deformation indexes and both non-invasive tissue characterization CMR parameters and CVF (Supplemental Table 1).

## Discussion

The main findings of our study were that: (i) cIBS is not significantly different in patients with severe symptomatic AS according to the presence or absence of LGE; (ii) ultrasonic reflectivity indexes have neither relation to non-invasive markers of myocardial ultrastructural remodeling, as assessed by multi-parametric CMR, nor to CVF at EMB. In accordance with current clinical practice, where multimodality echocardiography is unable to provide direct markers of tissue composition, we found that IBS is of no added value to discriminate the presence of myocardial fibrosis in this setting.

Despite our negative results, we are mostly in line with previous correlation studies, namely with those that investigate the usefulness of ultrasonic reflectivity indexes in patients with less than extensive grades of myocardial fibrosis [16]. Older invasive correlation studies have demonstrated stronger correlations between echo amplitude signals, as measurements of reflectivity, only for patients with increased myocardial collagen content, identified from the highest levels of hydroxyproline/leucine ratios obtained from EMB [27]. In a 2012 study [28] it was demonstrated that cIBS in the basal septum was related to both global and regional LV dysfunction, as assessed by myocardial velocities, deformation, and diastolic function indexes, in a subgroup of patients with metabolic syndrome but high fibrotic burden. This last categorization being performed according to serum levels (terciles) of procollagen peptides. In another clinical correlation study of patients with Kawasaki disease [17], cIBS values were found to be higher in patients with coronary dilations and aneurysms when compared with those with no coronary lesions and healthy controls. Though far from strong, these correlations improved along the phase of the disease as well, being higher in patients presenting at both the subacute and convalescent period. In one of the few studies investigating IBS imaging in patients with severe AS [12], there were good correlations between reflectivity indexes and MF at histopathology, and septal IBS was the best variable to discriminate patients with no symptoms and preserved EF vs. those with congestive heart failure and LVEF compromise, when indexed for pericardial values. Either way, correlation analysis was only assessed at patients submitted to AVR and EMB sampling, in this case with symptoms and subnormal LVEF, with extensive grades (> 25%) of interstitial MF. To the best of our knowledge, our is one of the few studies addressing the use of IBS in patients with severe AS, particularly with a relatively uniform group of symptomatic patients with classical high gradient phenotype and preserved LVEF. We compared IBS with LGE, native T1 and derived ECV, as these are proposed in combination, at a multiparametric CMR protocol, to better describe extracellular matrix remodeling and collagen content in this setting [7]. We further stratify the patients according to the presence of LV replacement fibrosis (LGE +) and made the correlation analysis for both global LV and basal anterior septum, this last one at a place supposed to be coincident with the ROI positioning at echocardiographic views. As detailed in methods, the group of 30 patients with LGE was randomly selected according to the presence of LGE at any LV location and we did not specifically make the assessment for patients with exclusive basal anterior septum LGE, as we thought this could have limited our analysis. However, we have recently published data from this same cohort of patients [29], showing that LGE was predominantly distributed in the basal and mid inferior and anterior interventricular septum, either as isolated mid-wall or combined with the junctional pattern. Either way, even considering that LGE does not necessarily represent the type of histological fibrosis that is assessed at EMB [7], it has been established that there is a correlation between LGE and the histological quantification of fibrosis at EMB [30]. From this, we supposed that patients with LGE represent a group with an increase burden of MF, and we hypothesized that this could mean distinct measurements for reflectivity indexes.

To further enhance the strength of a possible positive correlation of IBS with non-invasive markers, we quantified MF invasively at septal surgical EMB i.e., transaortic 0.5-10 mm sub valvular sample, making the same correlation analysis. As our quantification of MF at the gold-standard method is clearly less than extensive (median CVF below 10%), the lack of discriminatory power of IBS as a non-invasive method of fibrosis assessment in this cohort aligns with previous studies. Even so we should recognize that, particularly for segmental values of native T1 and ECV, conventional IBS estimation from a ROI sample at bidimensional echo images is probably not perfectly matching or representing the same area of the LV septum at CMR. Additionally, EMB not only involves endocardial tissue as it is also smaller than the 4 × 4 mm ROI dimension, positioned at the mid-septum. As said, dense subendocardial fibrosis was excluded from the quantification to counteract this issue and gather more mid-septum representativity. Finally, LGE quantification is for global LV replacement instead of histology derived MF, and this should be kept in mind for the correlation with IBS at a specific point of the interventricular septum. Strain derived IBS was neither related to non-invasive markers of myocardial structure nor to CVF, and this is probably related to the fact that cyclic variation of reflectivity is more a marker of contractility [13]. Even so, we could not find significant correlations between IBS and global and regional longitudinal deformation indexes at the additional correlation analysis.

The theoretical basis supporting the relation between myocardial tissue characterization and myocardial physical properties that define the interaction with ultrasonic waves may explain our results. Older studies have shown that collagen is a major determinant of echo intensity and ultrasonic backscatter expected to be sensitive to collagen type, postprocessing and organizational level [31]. However, several individual scattering elements within the myocardium, such as sarcomere sizes and lengths, mitochondrial content, myofiber architecture and disarray, but also interstitial space expansion and vessel density, may influence IBS signals [32]. In addition, functional parameters, which include contractile performance and loading conditions, may also influence tissue acoustic behaviour [12, 33]. We believe that our cohort is quite homogeneous in terms of AS severity and LV systolic function. Both groups of patients, with and without LGE at preoperative CMR, have classical AS phenotype and less than extensive grades of MF at histopathology, which could explain indistinct reflectivity indexes. Our estimation of ECV agrees with this last finding, being lower when compared with previous studies in AS cohorts [12, 34]. As collagen deposition, turnover, qualitative change, and organization determines extracellular matrix expansion, ECV would be expected to increase for more extensive grades of MF, this together influencing reflectivity indexes.

Despite being able to provide combined information towards valvular disease, global and regional LV function in patients with severe AS, echocardiography did not equally provide ultrastructural MF assessment by IBS. For more advanced LV disease stage in this setting, we might suppose that IBS may be of value in trying to identify patients with high fibrotic burden, eventually in some patients with preserved LVEF, whose prognosis would be deemed worse. As for myocardial strain, which is not capable to provide direct information towards ultrastructural composition, as also confirmed by our additional correlation, cyclic derived strain IBS was neither related to markers of fibrotic content.

EMB is both inappropriate and mainly feasible in already surgical referred patients, with sampling related limitations. On the other hand, CMR lacks echo´s availability, being expensive for the ever-increasing number of patients with severe AS. Hence, additional correlation studies are needed in trying to better assess IBS feasibility, reliability, and cut-off values for ultrastructural characterization. The final goal would be to develop ultrasound-based *echomics signatures*, as derived from reflectivity indexes, for non-invasive identification of MF at routine pre-operative echocardiography.

### Limitations

Technical details concerning IBS assessment are far from standardized. We were not able to use a defined protocol and we followed the measurements according to our echocardiographic equipment and post-processing software. For the sake of uniformization, we calibrated IBS for the pericardium to reduce the effects of distinct imaging setting such as depth, gain, compensation and temporal resolution, as recommended [23].

Reflectivity indexes, except strain derived IBS, were acquired at QRS-peak to improve the acoustic signal from distinct myocardial scatters, as in theory these are better apart from each other during diastole. Contrary to previous data assessing the usefulness of IBS in patients with AS [12] we did not evaluate the IBS variation and its cyclic variation index, as these were particularly related to LV systolic function instead of myocardial fibrotic content. Even so, we tried to assess myocardial ultrastructure with whole cardiac cycle absolute values of IBS from 2D strain.

We made this correlation analysis in a mostly uniform phenotype of patients with severe AS. Nevertheless, a control group of patients (non-severe AS and with no LV remodeling or dysfunction) could have been of value in trying to assess the possible distinct order of magnitude in terms of the acoustic measurements. In the same way, a group of patients with severe AS and extensive MF might have provided useful IBS cut-off values.

Finally, the presence of disarray at histology might have interfered with the present results, as the orientation of the myocardial elements relative to the angle of insonation (anisotropic effect) should be considered for reflectivity properties [11].

## Conclusions

Integrated backscatter and derived indexes are unrelated to MF in symptomatic patients with severe AS referred for surgical AVR. As there is no correlation between IBS and both non-invasive markers of replacement and interstitial fibrosis and CVF at histopathology, reflectivity indexes are deemed not suitable for ultrastructural tissue characterization, at least in this group of patients with less than extensive grades of MF.

### Supplementary Information

**Additional file 1. **Supplemental methods.

**Additional file 2. **Supplemental results.

**Additional file 3: Supplementary Figure 1.** Study flow-chart depicting how both group of patients, with and without late gadolinium enhancement (LGE), were selected for the analysis. SAVR – surgical aortic valve replacement; TTE – transthoracic echocardiography; CMR – cardiac magnetic resonance; LV – left ventricular; IBS – integrated backscatter; I.V. – intra-venous; TAVR – transcatheter aortic valve replacement; EMB – endomyocardial biopsy. *two patients with small areas of subendocardial LGE – ischemic scars, with no previous history of ischemic cardiomyopathy. **Supplementary Figure 2.** Masson´s trichrome whole slide image for MF quantification. Endocardial delineation is depicted (dotted line) to exclude dense endocardial fibrosis before automatic algorithm quantification. **Supplementary Figure 3.** A) Detailed view with specific colour coded tissue components at automatic algorithm of quantification – white is fibrosis; B) Example of an automatic quantification table displaying absolute areas and proportion of each tissue components.

**Additional file 4: Supplemental Table 1.** Spearman correlation analysis between GLS and basal interventricular septum longitudinal strain (ivs LS) and global and localized CMR tissue characterization parameters respectively. The correlation between ivs LS and collagen volume fraction at endomyocardial biopsy is also depicted. Abbreviations as in tables 1, 2, 3 and 4.

## Data Availability

All data exposed in this article was acquired from our institution, after obtaining informed consent from patients.

## Abbreviations

2D: Bidimensional
AP3C: Apical 3 Chamber View
AVR: Aortic Valve Replacement
AS: Aortic Stenosis
IBS: Integrated Backscatter
cIBS: calibrated IBS
CMR: Cardiac Magnetic Resonance
CVF: Collagen Volume Fraction
ECV: Extracellular Volume
EF: Ejection Fraction
EMB: Endomyocardial Biopsy
GLS: Global Longitudinal Strain
LGE: Late Gadolinium Enhancement
LV: Left Ventricle
MF: Myocardial Fibrosis
NT–proBNP: N–terminal B–type natriuretic peptide
PLAX: Parasternal long-axis view
TTE: Transthoracic Echocardiography
