# Supplementary material for: Is myocardial fibrosis appropriately assessed by calibrated and 2D strain derived integrated backscatter?
Source: Cardiovasc Ultrasound. 2023 Aug 12;21:14. doi: 10.1186/s12947-023-00311-x (PMC10422833; doi:10.1186/s12947-023-00311-x)
Supplement: Supplementary file 1 — Additional file 1. Supplemental methods. [file 12947_2023_311_MOESM1_ESM.docx]

**IS MYOCARDIAL FIBROSIS APPROPRIATELY ASSESSED BY CALIBRATED AND 2D STRAIN DERIVED INTEGRATED BACKSCATTER?**

**SUPPLEMENTAL MATERIAL – DETAILED METHODS**

**Corresponding Author**

Maria Rita Lima

Address: Av. Prof. Dr. Reinaldo dos Santos, 2790-134 Carnaxide, Lisbon, Portugal

Telephone: +351 21 043 1000

E-mail: mlima@chlo.min-saude.pt

**SUPPLEMENTAL METHODS**

**Study population and exclusion criteria**

Supplemental figure 1 details how we did select both groups of patients, with and without late gadolinium enhancement (LGE) at preoperative cardiac magnetic resonance (CMR) study. From the cohort of patients who were submitted to surgical aortic valve replacement and with appropriate EMB for myocardial fibrosis quantification, we divided the patients according to the presence or absence of LGE and made a random selection of 30 patients in each of the groups for the correlation analysis.

We excluded patients with congenital AS or previous diagnosis of sub/supra valvular aortic stenosis, concomitant severe non-aortic valve dysfunction, moderate and severe aortic regurgitation, previous cardiac surgery, active endocarditis, previous history of myocardial infarction, myocarditis, ischemic and non-ischemic cardiomyopathy including cardiac amyloidosis and other infiltrative diseases, chronic kidney disease with glomerular filtration rate below 30mL/min/1.73m^2^, non-cardiac inflammatory disease, active infection, under immunosuppressive and chronic anti-inflammatory therapy, under chemotherapy and with previous chest radiotherapy.

**Standard echocardiographic study – evaluation for aortic valve stenosis**

All patients underwent a comprehensive TTE in accordance with current guidelines [1].

As proposed [2] and for the quantification of AS, LV outflow tract diameter was measured on parasternal long-axis view and pulsed-wave and continuous-wave Doppler were used to record velocities across the LV outflow tract and aortic valve (AV), respectively. LV stroke volume index was calculated as LV outflow tract velocity time integral, obtained at 5-chamber apical view x LV outflow area/body surface area. Multiple TTE windows, including apical, subcostal, right parasternal and suprasternal, were assessed to obtain the highest well defined AV velocity signal, which was used to obtain the peak AV velocity and mean AV gradient, estimated using the Bernoulli equation. AV area was calculated with the continuity equation.

More than mild valvular regurgitation was quantified and semi-quantified using the proximal isovelocity surface area (PISA) method (including regurgitant volume and effective regurgitant orifice area), when appropriate, and *vena contracta* measurement, respectively [3].

All reported bidimensional and Doppler derived measurements were averaged over 3, or at least 5 cardiac cycles for patients in atrial fibrillation.

Ecocardiographic data collection and analysis was performed by experienced echocardiographers JA, RR and MJA.

**Cardiac Magnetic Resonance**

Post-contrast LGE imaging (10-12 short axis slices, 8mm thickness, 0mm gap) using Phase Sensitive Inversion Recovery (PSIR) (typical in-plane spatial resolution was 1.5mm x 1.5mm), was performed at 5 to 10 minutes after a bolus of 0.15mmol/Kg of gadobutrol, marked as Gadovist®, UK, and the inversion time was individually determined to null the normal myocardial signal. Native and postcontrast T1 mapping was performed using a *Modified Look-Locker Inversion recovery (MOLLI)* sequence in expiratory apnea, into three segments of the LV short-axis (base, mid and apex) before and 15 to 20 minutes after contrast injection, for ECV quantification, defined as ECV = (1-Htc) x [ΔR1_myocardium_]/[ΔR1_blood_][4], with the estimated Htc from the collected blood sample at the same day.

Post-processing and quantification were performed using a dedicated software (Circle Cardiovascular Imaging, CVI version 5.12, Calgary, Canada). LV volume, mass and EF were measured using standard volumetric techniques after endocardial and epicardial delineations in all end-diastolic and end-systolic phase short-axis images. The LV short-axis stack of post-contrast LGE was first assessed visually for its presence, followed by quantification, when present, also after endocardial and epicardial delineations. LGE was defined as areas of signal intensity ≥ 5 standard deviations from normal myocardium and was expressed as total mass and the percentage of total LV myocardial mass. Eventual presence of subendocardial LGE (not previously suspected) was excluded from the quantification.

Endo and epicardial delineations were also manually traced in all images of the three short-axis segments for pre- and post-gadolinium T1 estimation. We excluded 15% of the myocardial thickness at both endo and epicardial contours, in trying to minimize partial volume effects at blood pool interface, with potential T1 overestimation. Global myocardial T1 values were obtained and additional blood pool contours for each slice, avoiding papillary muscle inclusion, were performed. This enabled global ECV estimation after the introduction of the Htc level, as determined from individual blood samples collected at the same day of the examination. As per institutional protocol, our native T1 values were considered normal for the interval between 972 and 1029ms.

All patients, including those in atrial fibrillation, had controlled heart rates (between 50 and 90 beats per minute). All CMR results were read by two experienced readers (JA and AF) with Level 3 CMR accreditation by the European Association of Cardiovascular Imaging, blinded to both clinical and echocardiographic data.

***Myocardial Fibrosis Quantification at Histopathology***

The algorithm for automatic image processing, analysis and quantification algorithm was developed using the software platform *QuPath 0.3.0*. This widely used software, short for Quantitative Pathology, allows the development of tools for digital pathology image analysis, being specifically designed for WSIs without the need for cropping or down-sampling images [5] [6]. It allows the analysis of immunohistochemistry (brightfield or fluorescent) and haematoxylin and eosin (H&E) images, through a pattern recognition deep learning algorithm that distinguishes spatial and morphological features based on structures (classes) provided by the user [7]. Ultimately, the definition of specific thresholds for specific tissue compartment (for instance collagen fibers, vessel lumen, cardiomyocytes) and empty spaces, provides the base for quantification and proportion estimation (applying pixel counting and boundary detection, using specific colour values).

Our specific algorithm/classifier for the quantification of MF on *Masson´s* Trichrome brightfield images was developed as follows: 1) new project and training set created; 2) digital slides added to a training set; 3) colour stain vectors estimation – modal *RGB*; selection of objects for analysis, excluding dense endocardial fibrosis; 4) pixel classification loading according to specific classifier, previously created and tested (“*Monsta”*); specific task application algorithm for automatic analysis. For quantification, the total tissue component was split into fibrosis and a remaining part, containing cardiomyocytes, empty spaces, vessels, blood cells (detailed identification of fibrosis in white and empty spaces in orange/red). All components were assessed in pixels and a final table containing both absolute areas and correspondent proportions is displayed.

The whole analysis was performed on i*Mac 27*® (3.8 GHz, Intel Core i7, 8-GB RAM).

**REFERENCES FOR SUPPLEMENTAL MATERIAL_DETAILED METHODS**

1. Lang RM, Badano LP, Victor MA, Afilalo J, Armstrong A, Ernande L, et al. Recommendations for Cardiac Chamber Quantification by Echocardiography in Adults: An Update from the American Society of Echocardiography and the European Association of Cardiovascular Imaging. Journal of the American Society of Echocardiography [Internet]. 2015 [cited 2022 May 12];28:1-39.e14. Available from: http://www.onlinejase.com/article/S0894731714007457/fulltext

2. Baumgartner H, Hung J, Bermejo J, Chambers JB, Edvardsen T, Goldstein S, et al. Recommendations on the echocardiographic assessment of aortic valve stenosis: a focused update from the European Association of Cardiovascular Imaging and the American Society of Echocardiography. Eur Heart J Cardiovasc Imaging [Internet]. 2017;18:254–75. Available from: https://academic.oup.com/ehjcimaging/article-lookup/doi/10.1093/ehjci/jew335

3. Lancellotti P, Pibarot P, Chambers J, la Canna G, Pepi M, Dulgheru R, et al. Multi-modality imaging assessment of native valvular regurgitation: an EACVI and ESC council of valvular heart disease position paper. Eur Heart J Cardiovasc Imaging. 2022;

4. Messroghli DR, Moon JC, Ferreira VM, Grosse-Wortmann L, He T, Kellman P, et al. Clinical recommendations for cardiovascular magnetic resonance mapping of T1, T2, T2 and extracellular volume: A consensus statement by the Society for Cardiovascular Magnetic Resonance (SCMR) endorsed by the European Association for Cardiovascular Imagin. Journal of Cardiovascular Magnetic Resonance. 2017;19:1–24.

5. Bankhead P, Loughrey MB, Fernández JA, Dombrowski Y, McArt DG, Dunne PD, et al. QuPath: Open source software for digital pathology image analysis. Sci Rep [Internet]. 2017 [cited 2022 Nov 16];7. Available from: https://pubmed.ncbi.nlm.nih.gov/29203879/

6. Humphries MP, Maxwell P, Salto-Tellez M. QuPath: The global impact of an open source digital pathology system. Comput Struct Biotechnol J [Internet]. 2021 [cited 2022 Oct 11];19:852–9. Available from: https://pubmed.ncbi.nlm.nih.gov/33598100/

7. Eckstein J, Renner A, Zittermann A, Fink T, Sohns C, Niehaus K, et al. Impact of left atrial appendage fibrosis on atrial fibrillation in patients following coronary bypass surgery. Clin Cardiol [Internet]. 2022 [cited 2022 Oct 11];45:1029–35. Available from: https://onlinelibrary.wiley.com/doi/10.1002/clc.23884
