# Supplementary material for: Is myocardial fibrosis appropriately assessed by calibrated and 2D strain derived integrated backscatter?
Source: Cardiovasc Ultrasound. 2023 Aug 12;21:14. doi: 10.1186/s12947-023-00311-x (PMC10422833; doi:10.1186/s12947-023-00311-x)

**IS MYOCARDIAL FIBROSIS APPROPRIATELY ASSESSED BY CALIBRATED AND 2D STRAIN DERIVED INTEGRATED BACKSCATTER?**

**SUPPLEMENTAL MATERIAL – RESULTS**

**Corresponding Author**

Maria Rita Lima

Address: Av. Prof. Dr. Reinaldo dos Santos, 2790-134 Carnaxide, Lisbon, Portugal

Telephone: +351 21 043 1000

E-mail: mlima@chlo.min-saude.pt

**SUPPLEMENTAL RESULTS**

**Integrated backscatter in both group of patients, LGE + vs. LGE –**

cIBS and 2D strain derived IBS in patients with and without LGE at pre-operative CMR.


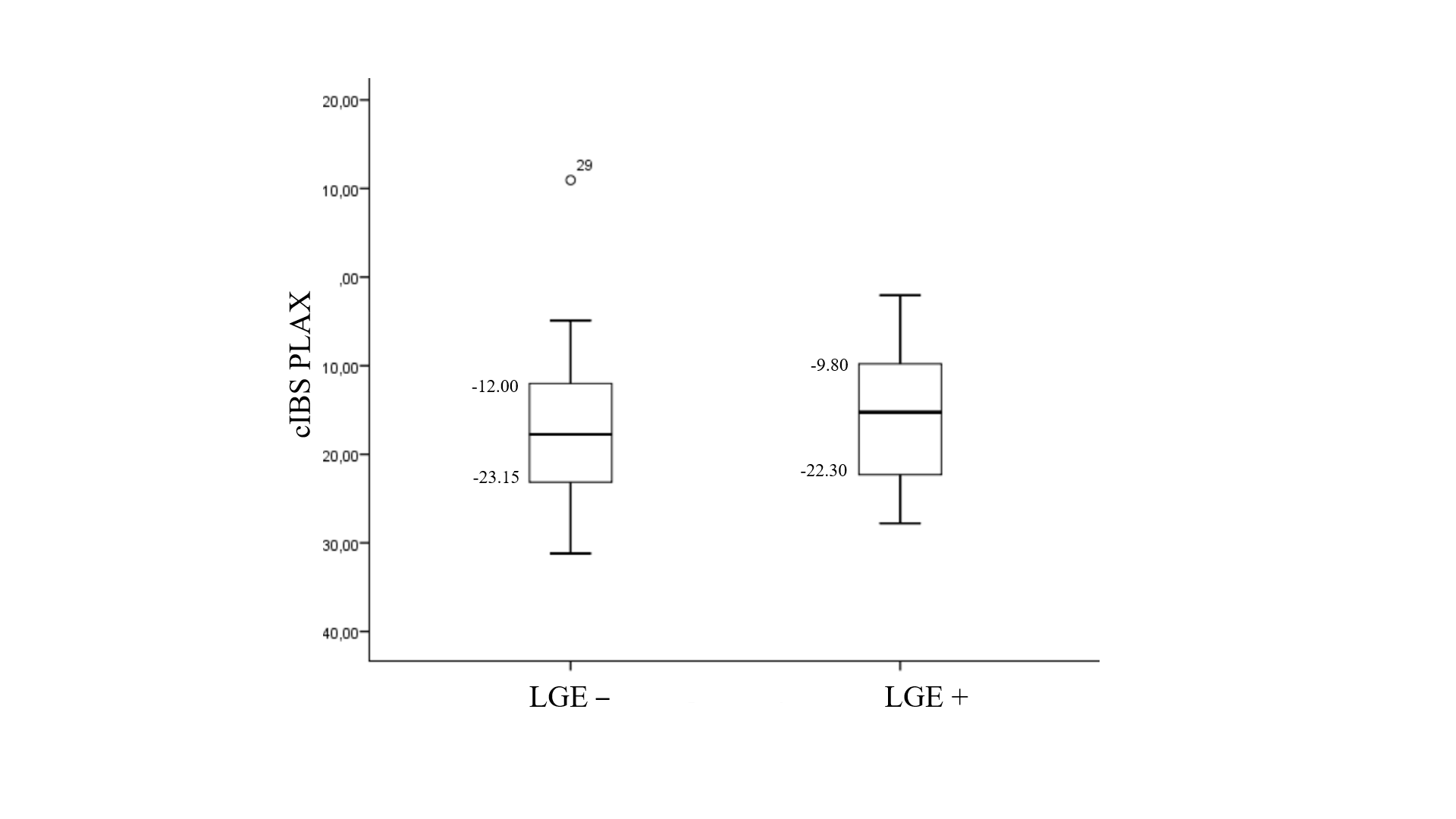


–17.75 (–31.2–10.95) vs. –14.45 (–27.8– –2.05), p=0.290


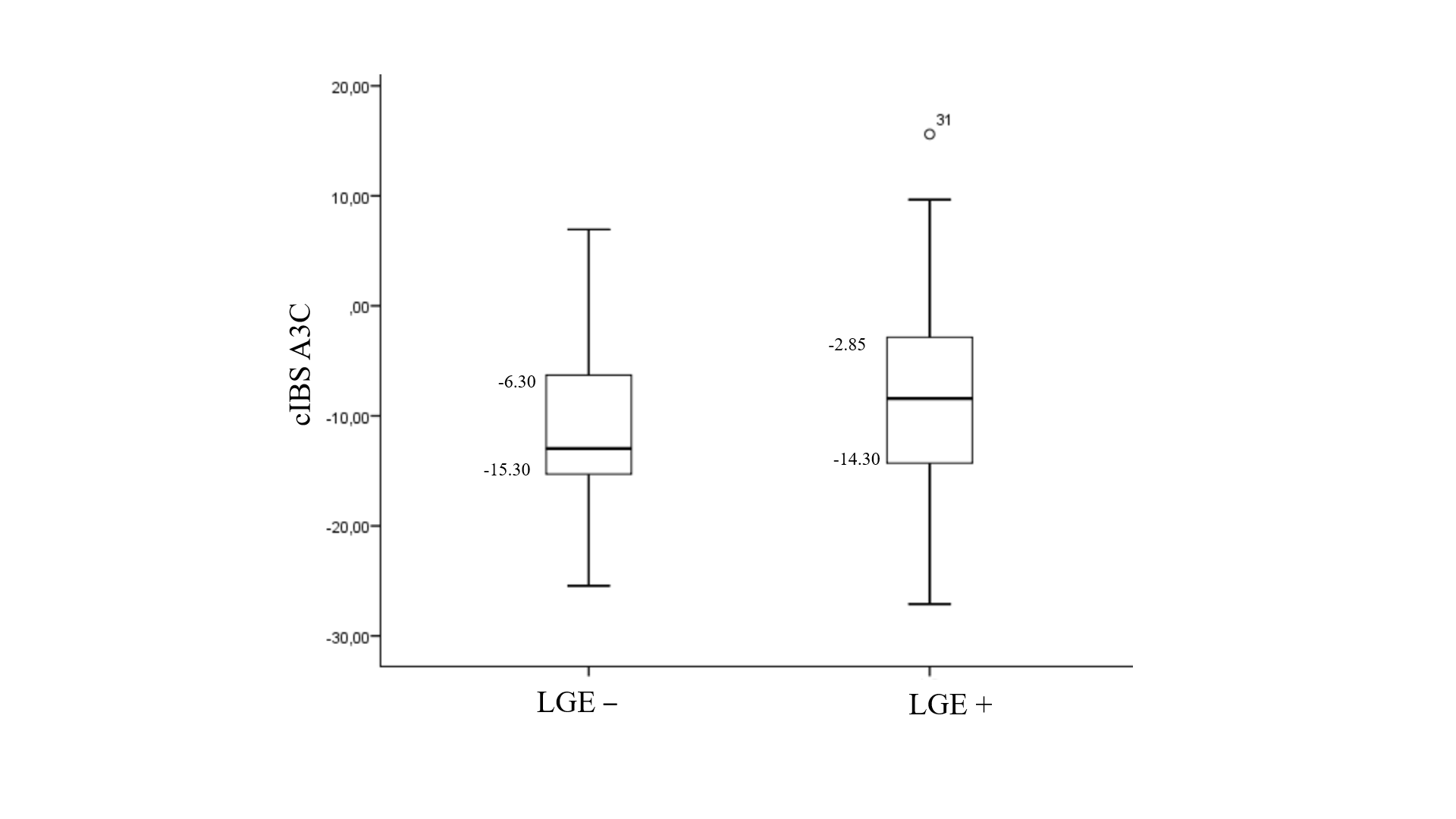


–11.28±7.91 vs. –8.13±9.72, p=0.189


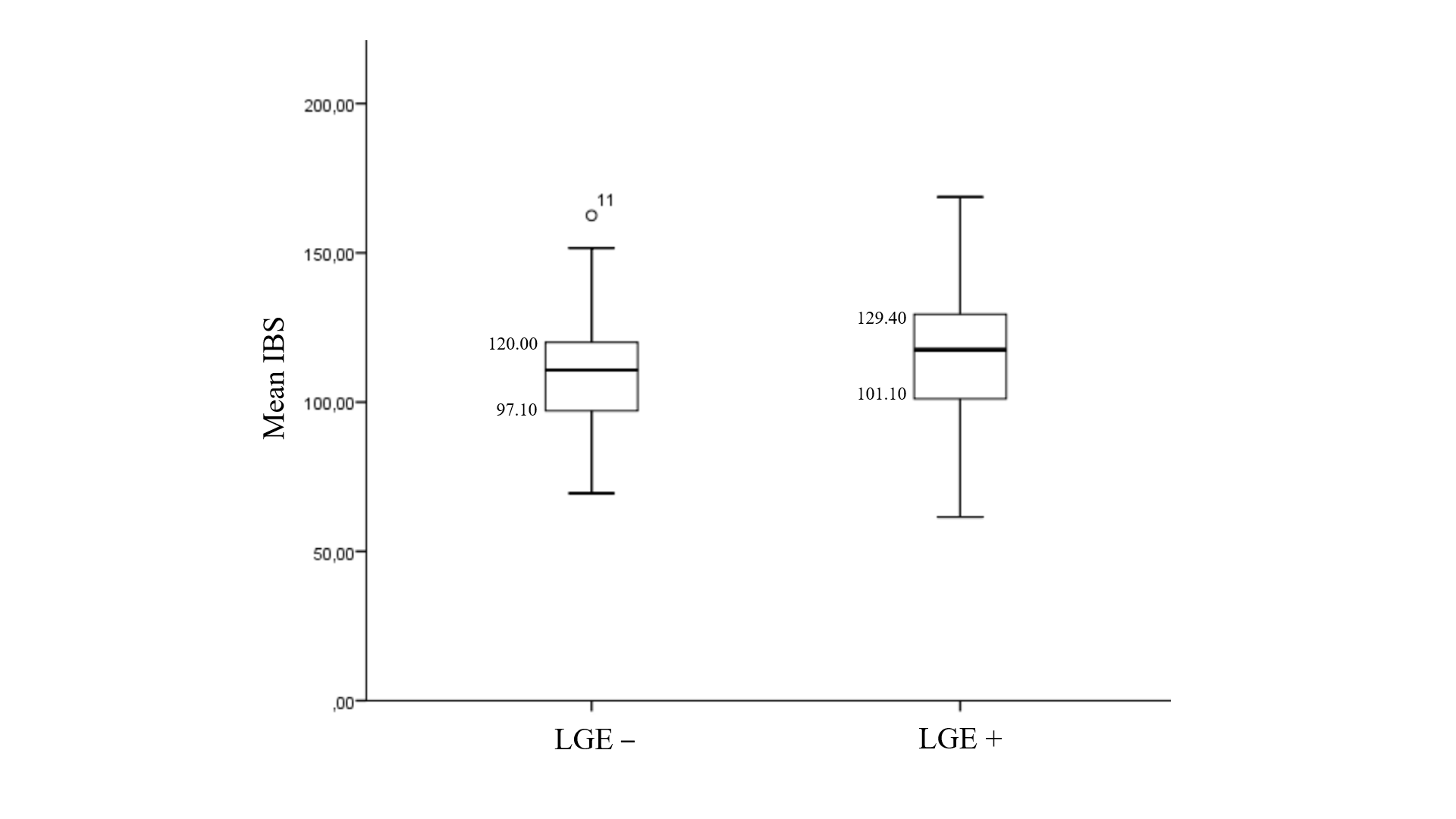


110.55±21.22 vs. 117.43±23.83, p=0.241

**Integrated backscatter: correlations with invasive and non-invasive assessment of fibrosis**

*Correlation between cIBS from PLAX and AP3C views*

cIBS values were significantly lower when calculated from AP3C view [–9.17±9.45 vs. –17.45 (–31.2–10.95), p<0.001], with no correlation between them (r=0.141, p=284).


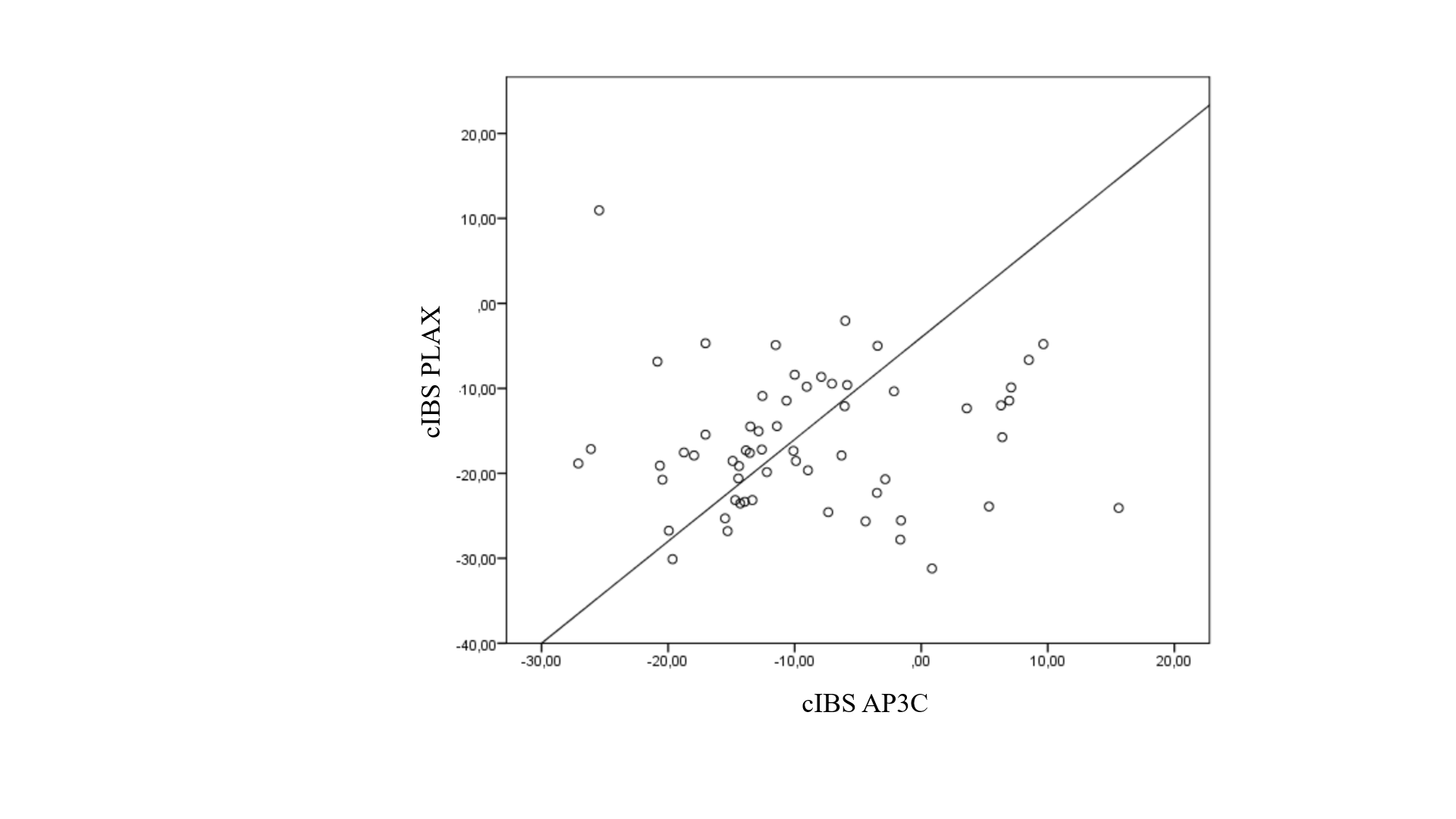


*Bland-Altman plots for both intra and interobserver assessment of IBS measurements*

Intra-observer agreement: the mean difference of measurements regarding mean IBS was -9.125 ± 21.951 (95% CI -52.150- 33.899; p=0.130). The mean difference of cIBS was -0.5997±11.407 (95% CI -22.957-21.758; p=0.842) in PLAX and 8.087±15.849 (95% CI -39,152-22,977; p=0.068) in A3C view.

*Intra-observer variability of mean IBS*


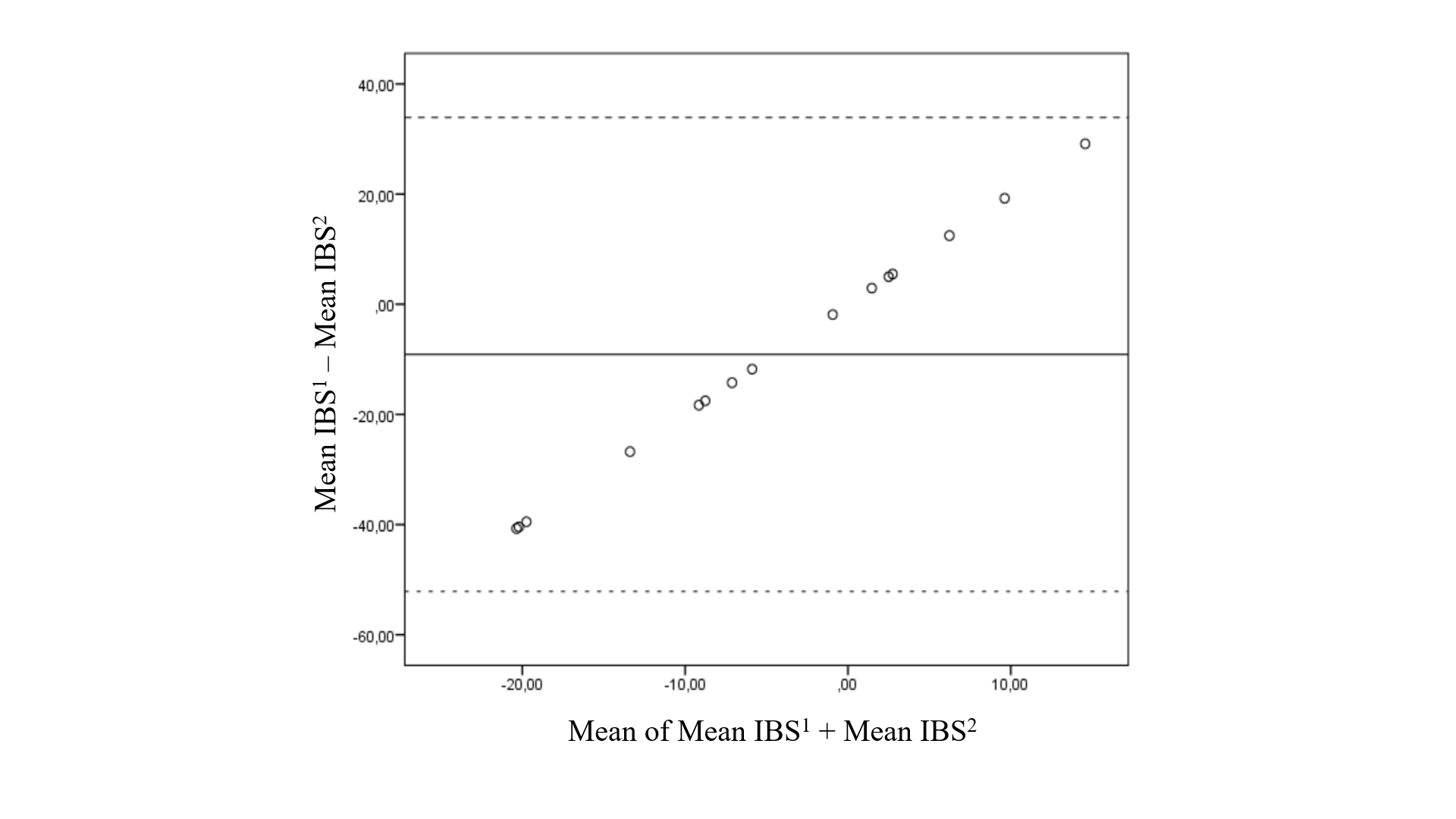


*Bland-Altman plot, representing the agreement between both strain derived IBS measurements. The mean difference between both measurements was not significantly different from zero (the value is included in the mean difference CI), which shows a good agreement. Solid line shows mean; dotted lines denote ±1.96 SD. This legend applies to all the other Bland-Altman plots.

*Intra-observer variability of cIBS at PLAX view*


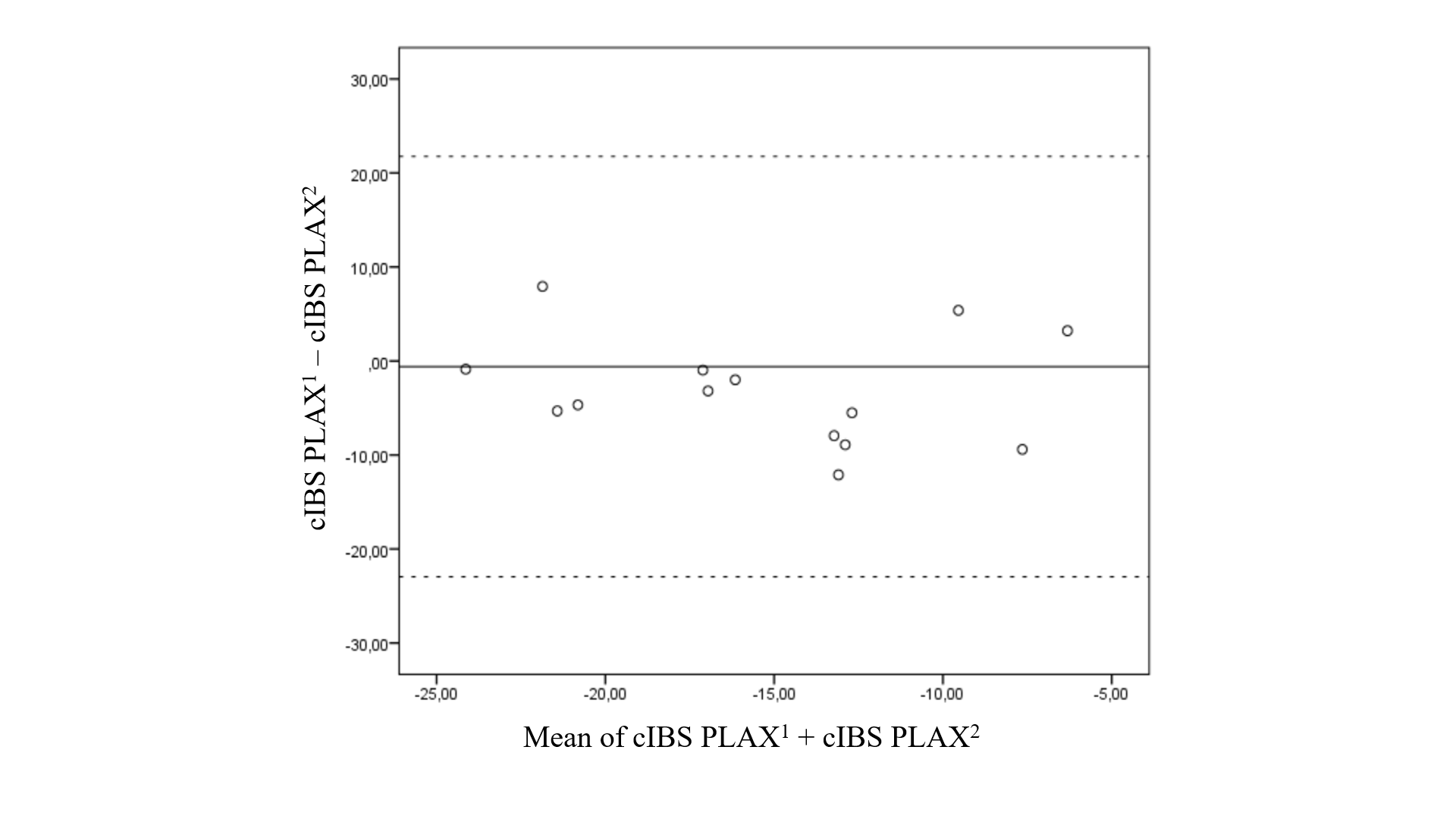


*Intra-observer variability of cIBS at A3C view*


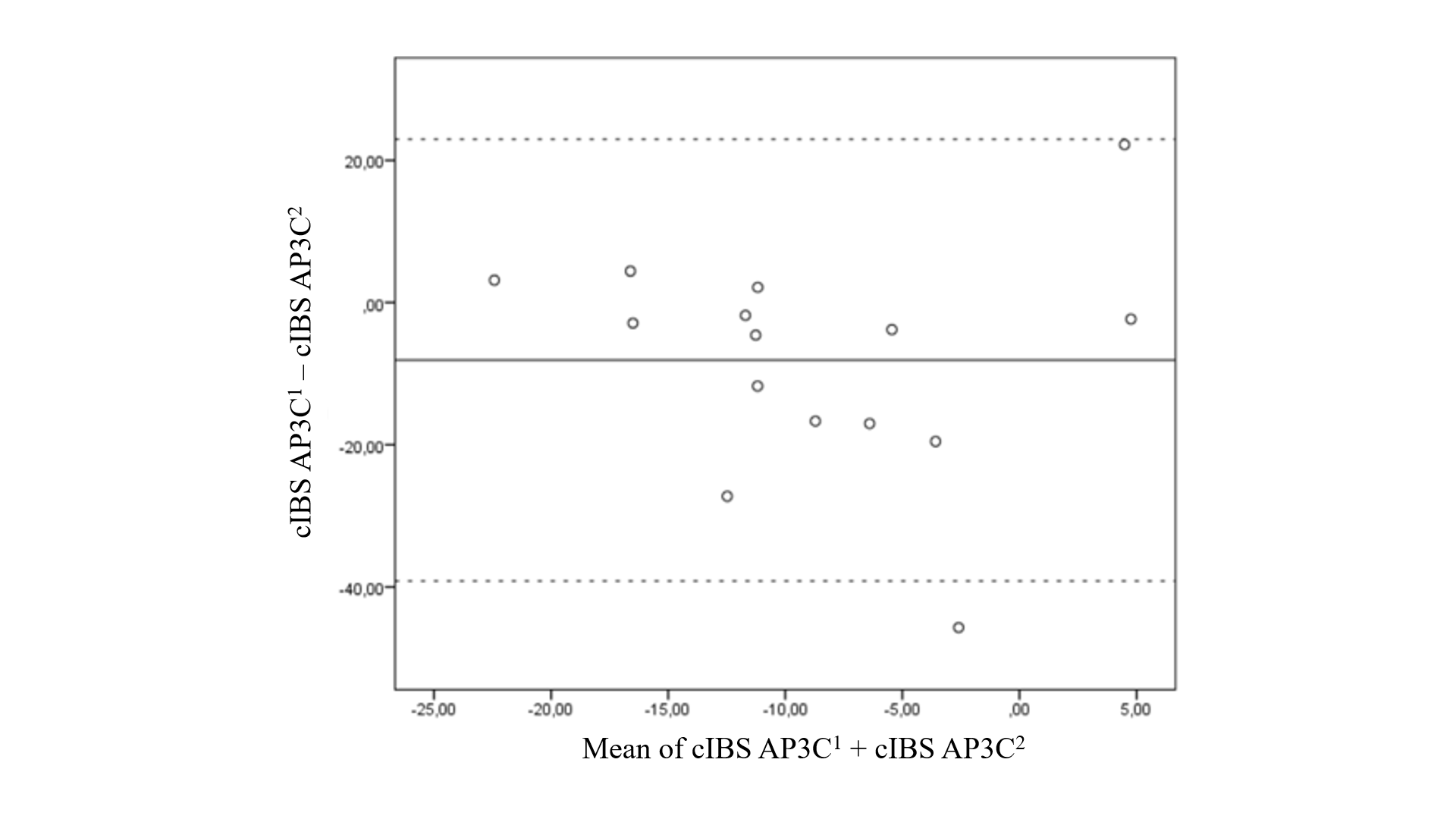


Interobserver agreement: The mean difference of measurements regarding mean IBS was 4.613±20.972 (95% CI -36.491–45.719; p=0.03). The mean difference of cIBS in PLAX views was 0.432±7.599 (95% CI -14.462–15.327; p=0.091) and the mean difference of cIBS in A3C views was 0.706±10.020 (95% CI -18.933–20.347; p=0.660).

*Interobserver variability of mean IBS*


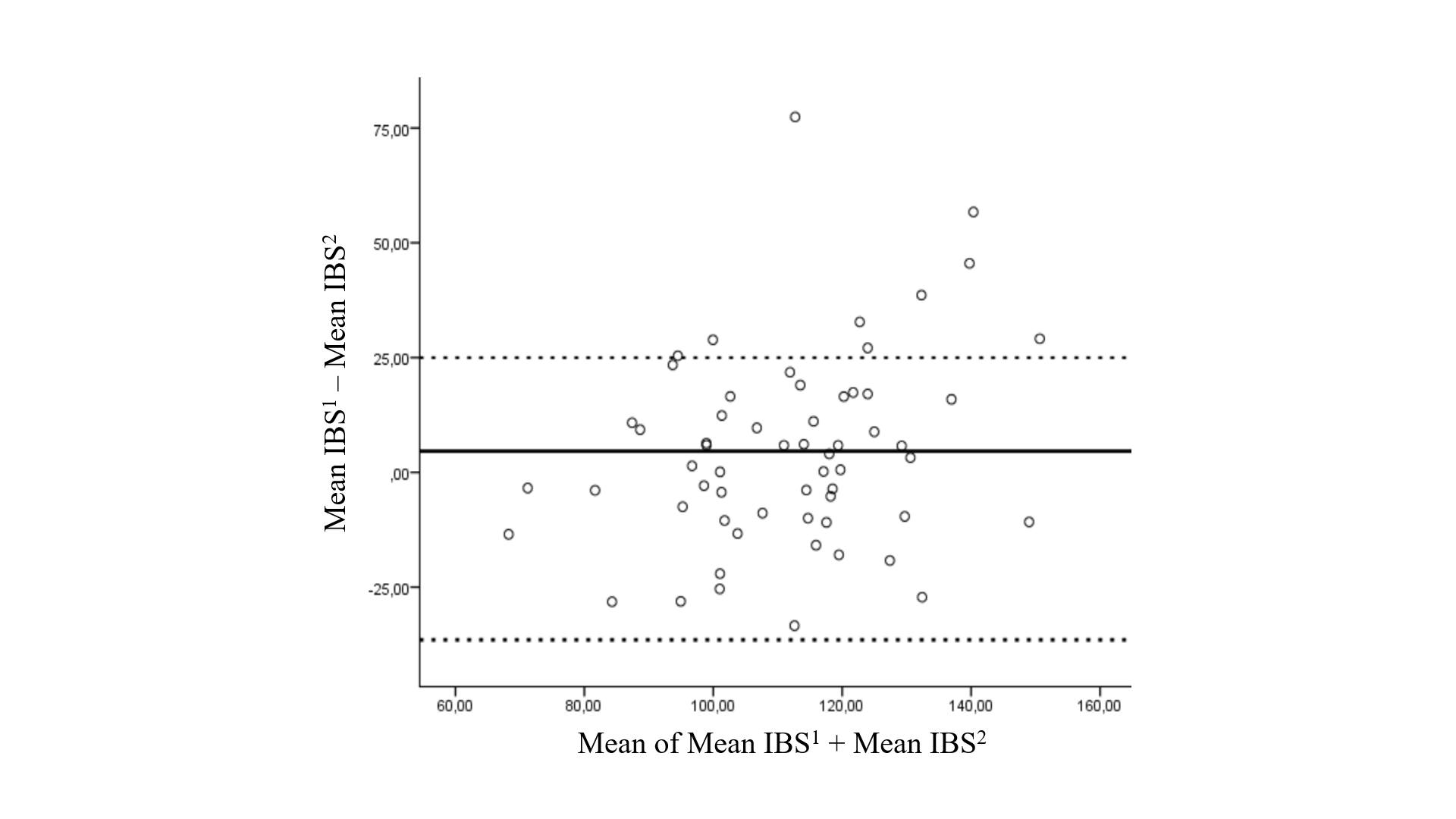


*Interbserver variability of cIBS at PLAX view*


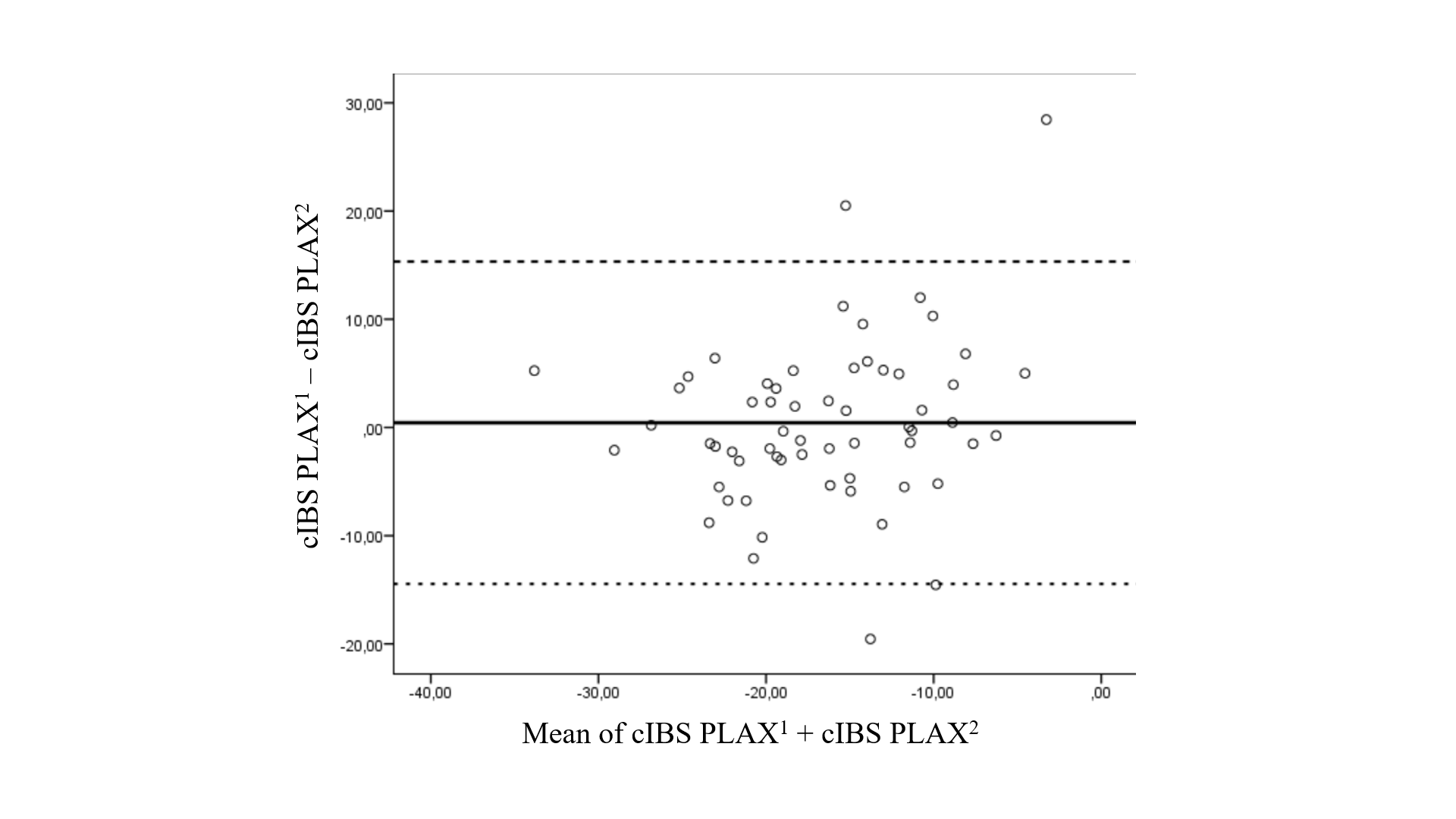


*Interbserver variability of cIBS at A3C view*


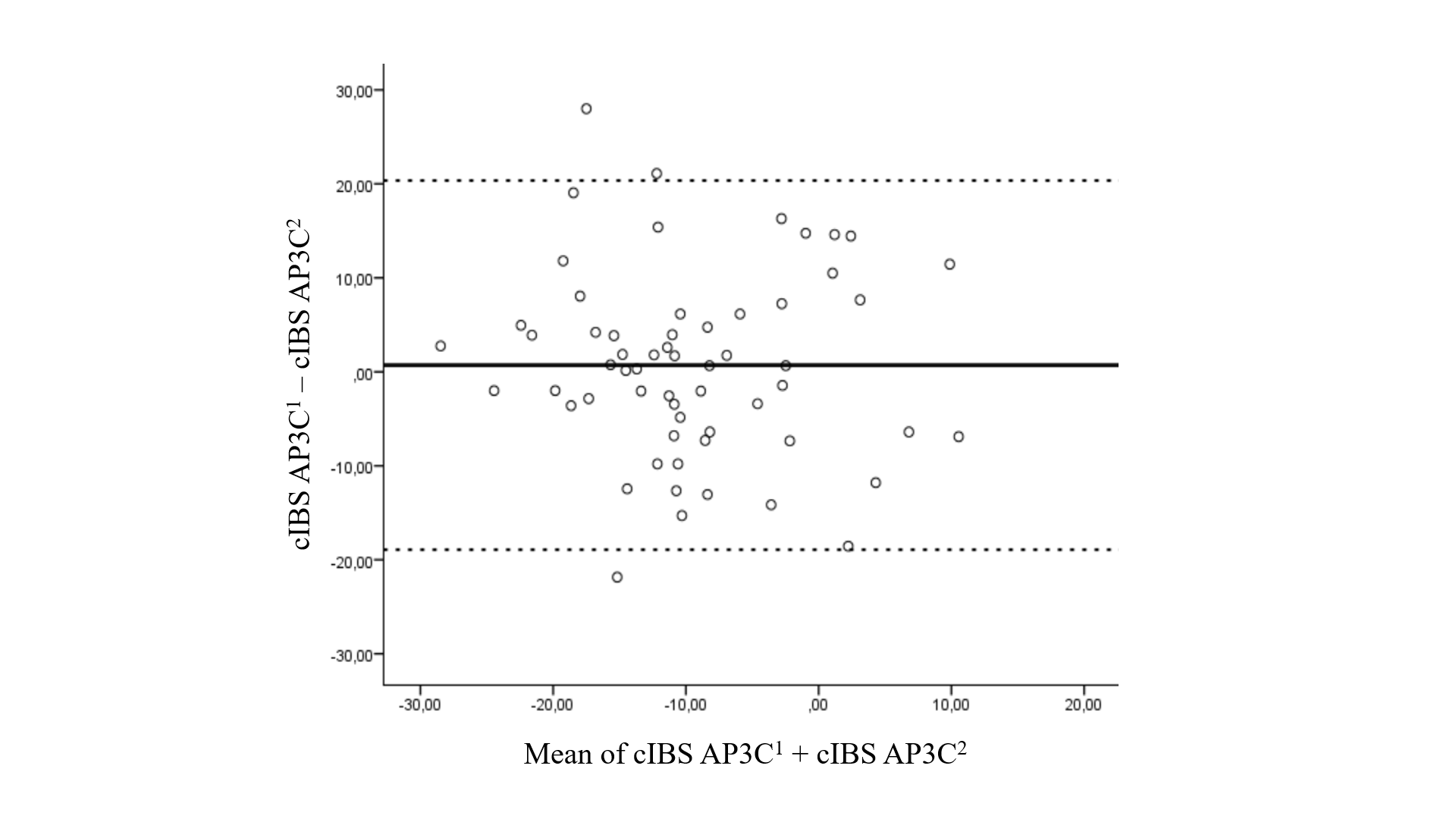


**Correlations between reflectivity indexes and CMR derived measurements (native T1, ECV, LGE mass) and invasive quantification of MF at histopathology**

*cIBS at PLAX*


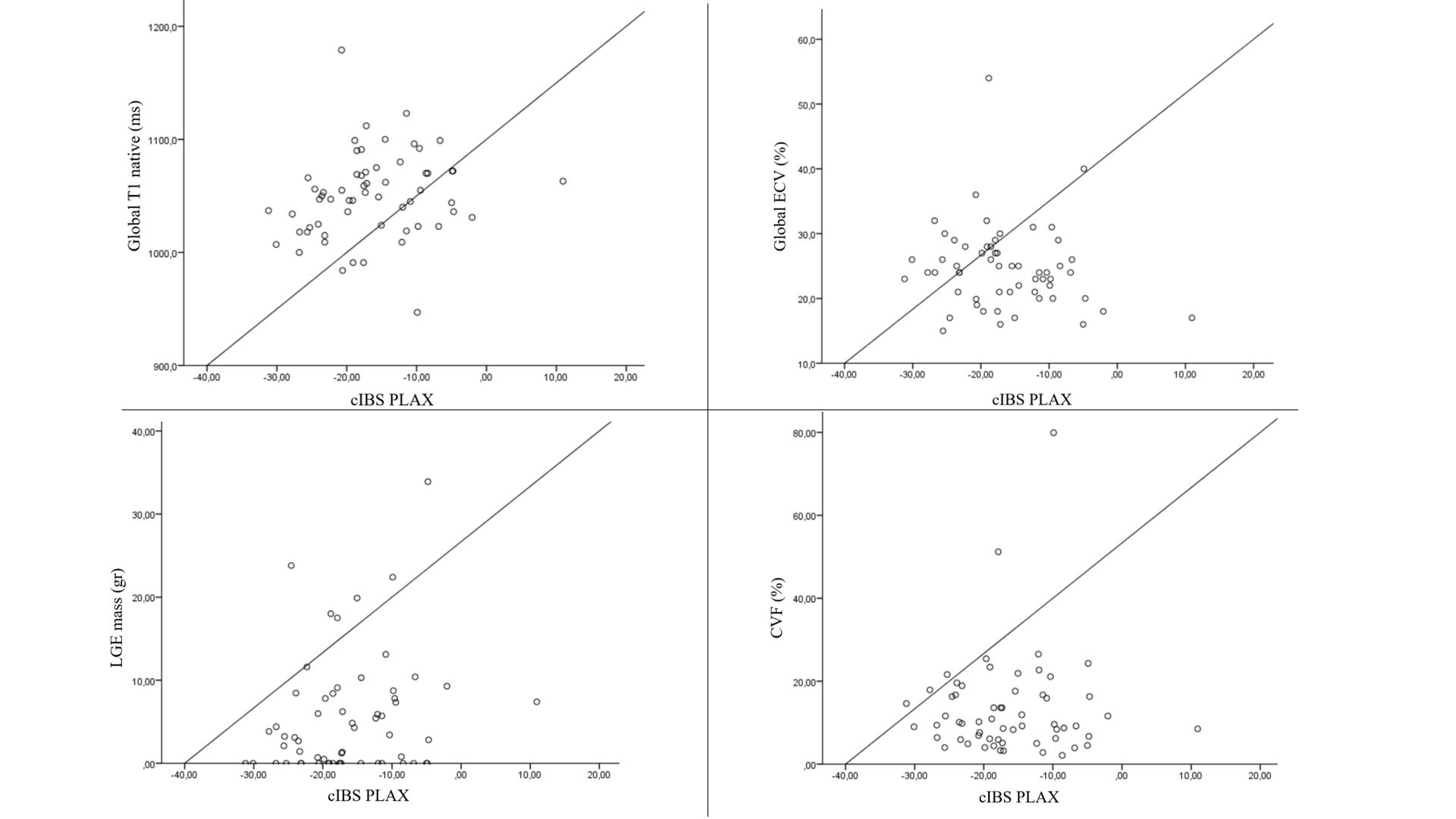


*cIBS at AP3C*


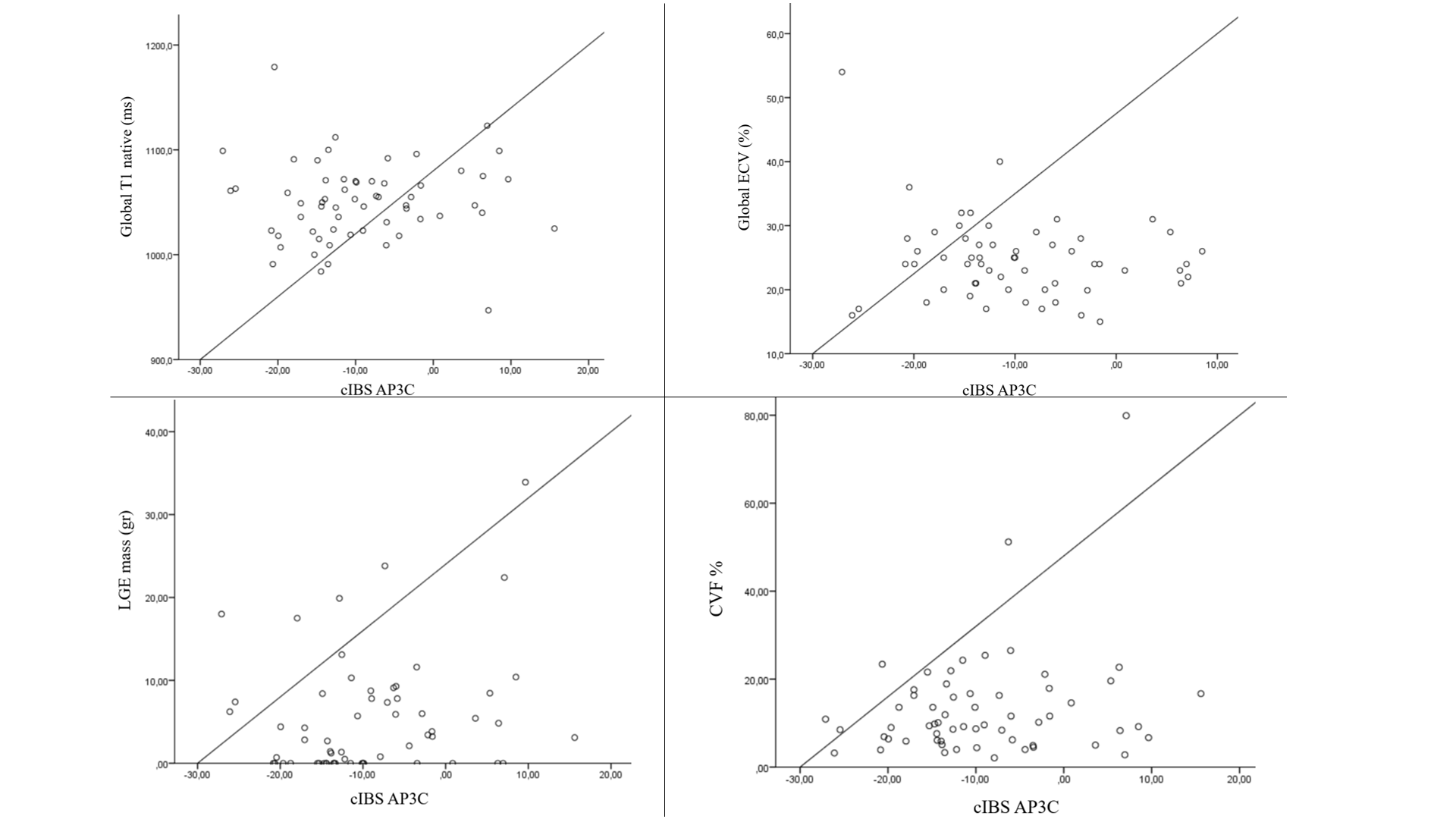


*Strain derived IBS*


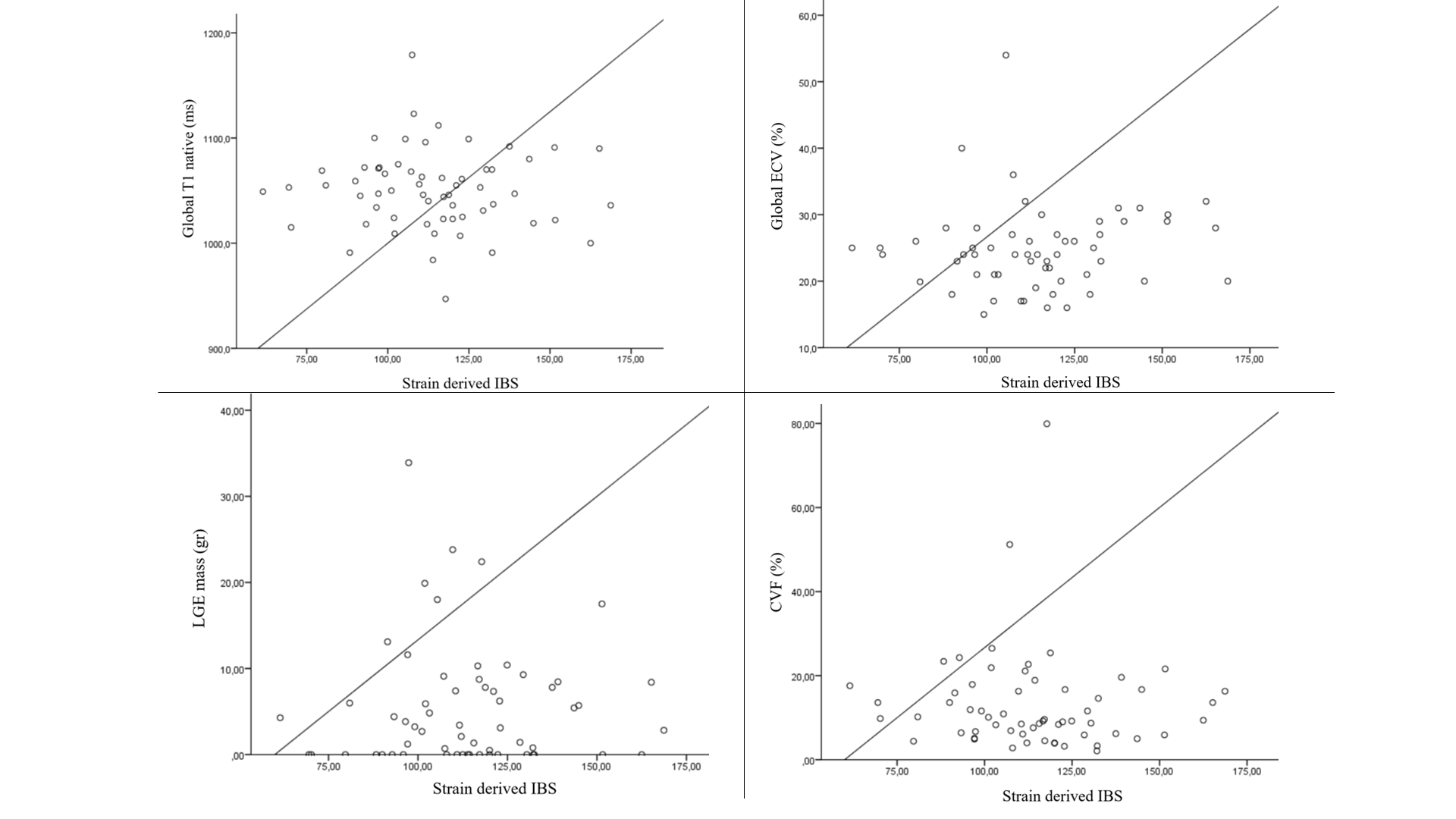

Supplement: Supplementary file 2 — Additional file 2. Supplemental results. [file 12947_2023_311_MOESM2_ESM.docx]
