# Supplementary material for: Is myocardial fibrosis appropriately assessed by calibrated and 2D strain derived integrated backscatter?
Source: Cardiovasc Ultrasound. 2023 Aug 12;21:14. doi: 10.1186/s12947-023-00311-x (PMC10422833; doi:10.1186/s12947-023-00311-x)

**IS MYOCARDIAL FIBROSIS APPROPRIATELY ASSESSED BY CALIBRATED AND 2D STRAIN DERIVED INTEGRATED BACKSCATTER?**

**SUPPLEMENTAL MATERIAL – FIGURE LEGENDS**

**Corresponding Author**

Maria Rita Lima

Address: Av. Prof. Dr. Reinaldo dos Santos, 2790-134 Carnaxide, Lisbon, Portugal

Telephone: +351 21 043 1000

E-mail: mlima@chlo.min-saude.pt

***SUPPLEMENTARY FIGURE 1.*** *Study flow-chart depicting how both group of patients, with and without late gadolinium enhancement (LGE), were selected for the analysis. SAVR – surgical aortic valve replacement; TTE – transthoracic echocardiography; CMR – cardiac magnetic resonance; LV – left ventricular; IBS – integrated backscatter; I.V. – intra-venous; TAVR – transcatheter aortic valve replacement; EMB – endomyocardial biopsy. *two patients with small areas of subendocardial LGE – ischemic scars, with no previous history of ischemic cardiomyopathy.*

***SUPPLEMENTARY FIGURE 2.*** *Masson´s trichrome whole slide image for MF quantification. Endocardial delineation is depicted (dotted line) to exclude dense endocardial fibrosis before automatic algorithm quantification.*

***SUPPLEMENTARY FIGURE 3.*** *A) Detailed view with specific colour coded tissue components at automatic algorithm of quantification – white is fibrosis; B) Example of an automatic quantification table displaying absolute areas and proportion of each tissue components.*

***
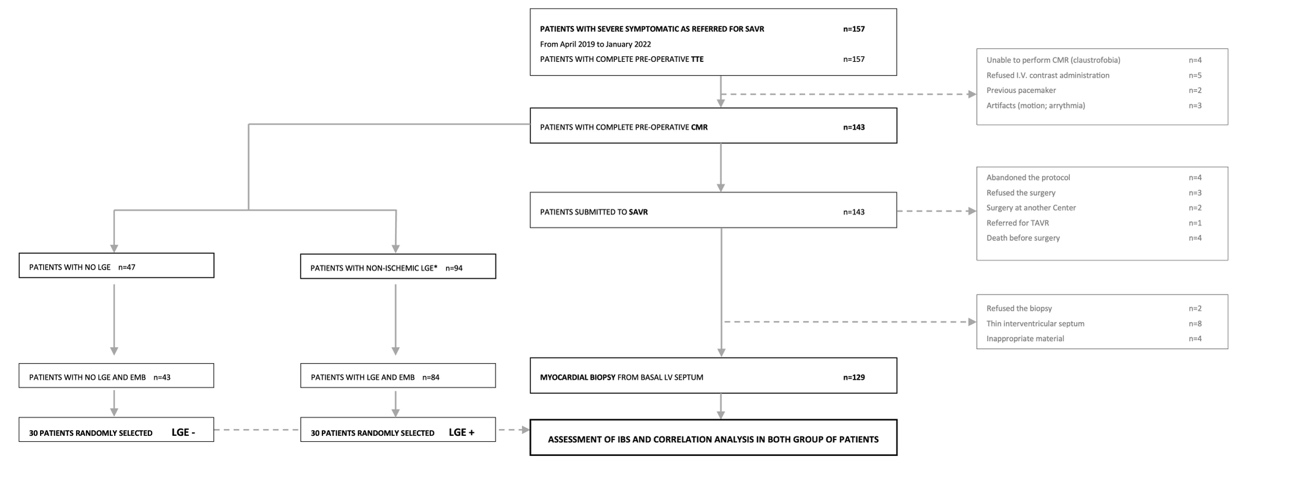
SUPPLEMENTARY FIGURE 1.***

***SUPPLEMENTARY FIGURE 2.***


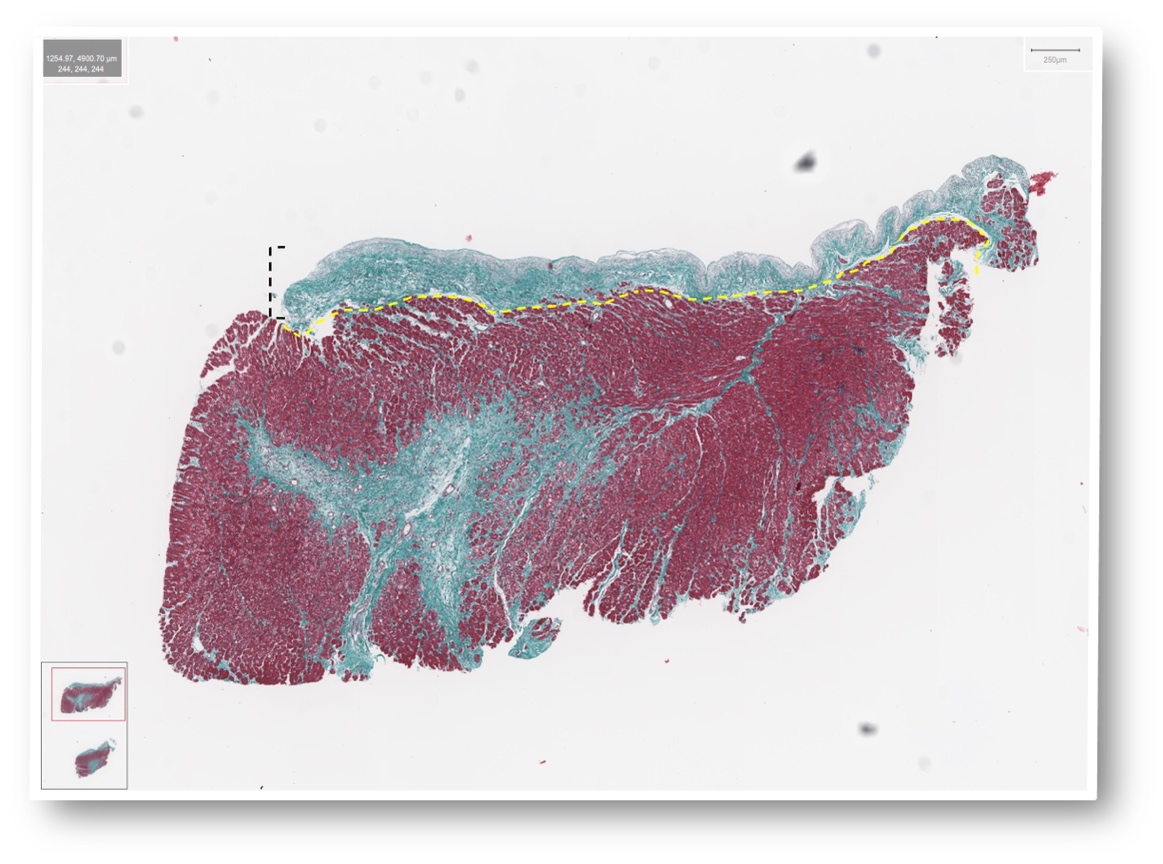


***SUPPLEMENTARY FIGURE 3.***


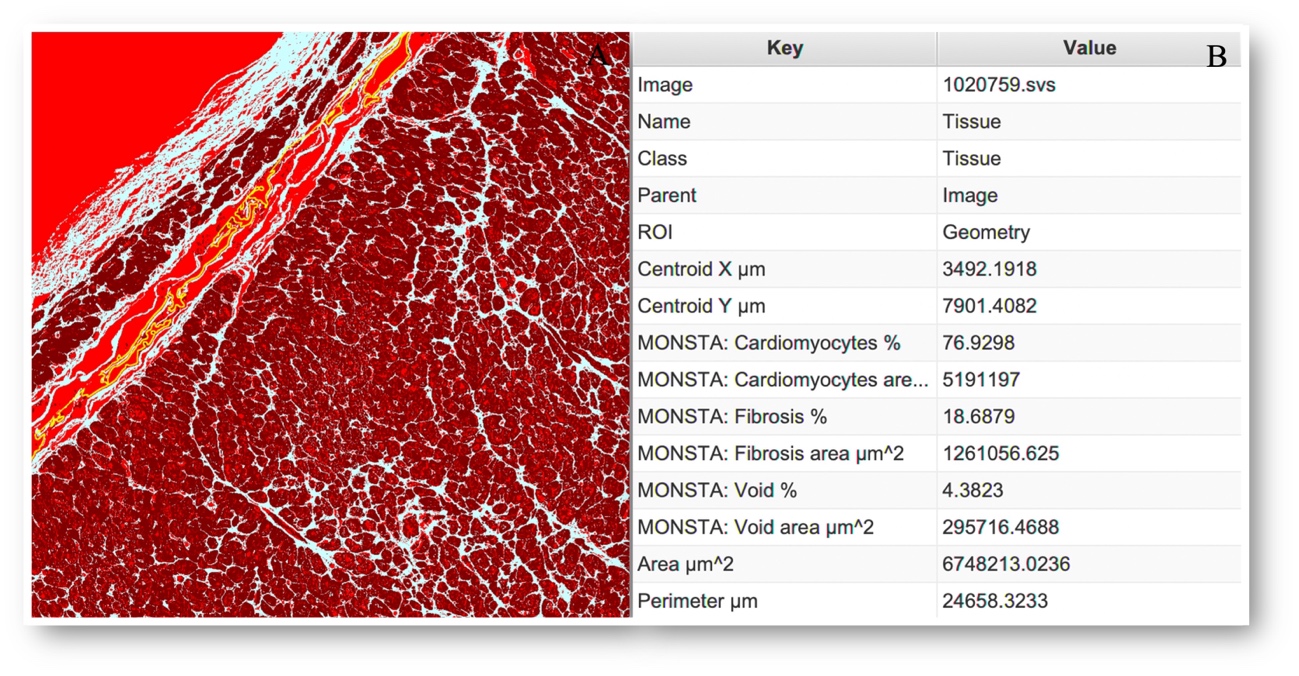

Supplement: Supplementary file 3 — Additional file 3: Supplementary Figure 1. Study flow-chart depicting how both group of patients, with and without late gadolinium enhancement (LGE), were selected for the analysis. SAVR – surgical aortic valve replacement; TTE – transthoracic echocardiography; CMR – cardiac magnetic resonance; LV – left ventricular; IBS – integrated backscatter; I.V. – intra-venous; TAVR – transcatheter aortic valve replacement; EMB – endomyocardial biopsy. *two patients with small areas of subendocardial LGE – ischemic scars, with no previous history of ischemic cardiomyopathy. Supplementary Figure 2. Masson´s trichrome whole slide image for MF quantification. Endocardial delineation is depicted (dotted line) to exclude dense endocardial fibrosis before automatic algorithm quantification. Supplementary Figure 3. A) Detailed view with specific colour coded tissue components at automatic algorithm of quantification – white is fibrosis; B) Example of an automatic quantification table displaying absolute areas and proportion of each tissue components. [file 12947_2023_311_MOESM3_ESM.docx]
