# Supplementary material for: Is myocardial fibrosis appropriately assessed by calibrated and 2D strain derived integrated backscatter?
Source: Cardiovasc Ultrasound. 2023 Aug 12;21:14. doi: 10.1186/s12947-023-00311-x (PMC10422833; doi:10.1186/s12947-023-00311-x)
Supplement: Supplementary file 4 — Additional file 4: Supplemental Table 1. Spearman correlation analysis between GLS and basal interventricular septum longitudinal strain (ivs LS) and global and localized CMR tissue characterization parameters respectively. The correlation between ivs LS and collagen volume fraction at endomyocardial biopsy is also depicted. Abbreviations as in tables 1, 2, 3 and 4. [file 12947_2023_311_MOESM4_ESM.docx]

**IS MYOCARDIAL FIBROSIS APPROPRIATELY ASSESSED BY CALIBRATED AND 2D STRAIN DERIVED INTEGRATED BACKSCATTER?**

**SUPPLEMENTAL RESULTS – SUPPLEMENTAL TABLE**

**Corresponding Author**

Maria Rita Lima

Address: Av. Prof. Dr. Reinaldo dos Santos, 2790-134 Carnaxide, Lisbon, Portugal

Telephone: +351 21 043 1000

E-mail: mlima@chlo.min-saude.pt

**SUPPLEMENTAL TABLE 1**

|  | ***r*** | ***p-value*** |
| --- | --- | --- |
|  | ***ECOCARDIOGRAPHY – GLS*** | |
| ***CMR – Tissue characterization*** | | |
| LGE | 0.324 | **0.012** |
| Global T1 | 0.248 | 0.056 |
| Global ECV | -0.30 | 0.822 |
|  | ***ECOCARDIOGRAPHY – IVS LS*** | |
| ***CMR – Tissue characterization*** | | |
| LGE | 0.387 | **0.002** |
| ivs T1 | 0.055 | 0.678 |
| ivs ECV | -0.027 | 0.835 |
| ***Histology at Endomyocardial Biopsy*** | | |
| CVF | 0.075 | 0.569 |

***Supplemental table 1***. *Spearman correlation analysis between GLS and basal interventricular septum longitudinal strain (ivs LS) and global and localized CMR tissue characterization parameters respectively. The correlation between ivs LS and collagen volume fraction at endomyocardial biopsy is also depicted. Abbreviations as in tables 1, 2, 3 and 4.*
